# Conflict resolution in the Eriksen flanker task: Similarities and differences to the Simon task

**DOI:** 10.1371/journal.pone.0214203

**Published:** 2019-03-28

**Authors:** Ronald Hübner, Lisa Töbel

**Affiliations:** Department of Psychology, Universität Konstanz, Konstanz, Germany; Brunel University London, UNITED KINGDOM

## Abstract

In the Eriksen flanker task as well as in the Simon task irrelevant activation produces a response conflict that has to be resolved by mental control mechanisms. Despite these similarities, however, the tasks differ with respect to their delta functions, which express how the congruency effects develop with response time. The slope of the delta function is mostly positive for the flanker task, but negative for the Simon task. Much effort has been spent to explain this difference and to investigate whether it results from task-specific control. A prominent account is that the temporal overlap between irrelevant and relevant response activation is larger in the flanker task than in the Simon task. To test this hypothesis, we increased the temporal distance in a flanker task by presenting the flankers ahead of the target. This not only produced negatively sloped delta functions but also caused reversed congruency effects. We also conducted a Simon-task experiment in which we varied the proportion of congruent stimuli. As a result, the delta function was negatively sloped only if the proportion was low. These results demonstrate that a long temporal distance is necessary but not sufficient for observing negatively sloped delta functions. Finally, we modeled the data with drift-diffusion models. Together, our results show that differently sloped delta functions can be produced with both tasks. They further indicate that activation suppression is an important control mechanism that can be adapted rather flexibly to the control demands.

## Introduction

The ability to respond to stimuli depending on the current goal is an essential characteristic of human performance. An important prerequisite for such goal-directed behavior are mental control mechanisms that enable persons to respond to relevant stimuli or stimulus features and to prevent responding to irrelevant ones. Such control can be difficult, because, due to learning, stimulus features might automatically activate their associated responses. If these responses are different from those required for reaching the current goal, a response conflict occurs, which has to be resolved. For investigating involved conflict-resolution and control mechanisms, several *conflict paradigms* have been developed, such as the Stroop task [1, 2], the Eriksen flanker task [3, 4], and the Simon task [5–7]. In these paradigms, task-irrelevant stimuli or features are used to induce a response conflict. The irrelevant information, which is presented along with task-relevant information, is either response compatible, or response incompatible. The difference in performance between respective trials is called *congruency effect*, and the size and characteristics of this effect reflect the efficiency and limits of selectivity, and provide valuable information about the mechanisms involved in controlling the conflict.

With respect to mean performance, the mentioned conflict paradigms produce similar congruency effects. That is, response times (RTs) and error rates are larger for incongruent stimuli than for congruent ones. However, if one analyses the data in more detail and also considers RT distributions (e.g., [8]), then there are characteristic differences between the paradigms (for an overview, see [9]). One prominent approach in this respect is to consider how the congruency effect varies across latencies. For this objective, cumulative RT distributions are computed for correct responses to congruent and incongruent stimuli, respectively. The difference between these two distribution functions for a given percentile then represents the congruency effect at a certain latency, which is usually defined as the mean of the two percentile RTs. For example, let us assume that at the 50% percentile the RT for congruent stimuli is 410 *ms*, whereas that for incongruent stimuli is 450 *ms*. Then one would say that the there was a congruency effect of 40 *ms* at an RT of 430 *ms*. If one calculates these values for different percentiles, then one obtains a so-called *delta function*, also termed *delta plot* [10, 11], showing how the size of the congruency effect varies with RT. By examining such functions it has been found that the effect size in the latencies increases with RT for the flanker task (e.g., [12]), but decreases for the Simon task (e.g., [11]).

In view of these results, some researchers have hypothesized that the Simon task is special and involves specific control mechanisms (e.g., [9, 13]). Others, however, have questioned that this is the case (e.g., [14]). Moreover, Ulrich, Schröter [15] recently developed a *Diffusion Model for Conflict tasks* (DMC), which suggests that differently sloped delta functions can result from a single mechanism. According to this model, the slope and form of delta functions largely depend on the time to which irrelevant response activation occurs, relative to the start of relevant activation. The slope is positive or negative, at least in the last section (slow responses) of the function, depending on whether irrelevant activation occurs relatively late or early, respectively, during the course of response selection.

Because it is an important question which mental control processes are involved in a specific conflict task, the aim of the present study was to investigate in detail the control mechanisms for the Eriksen flanker task and to which extent they differ from those for the Simon task. Our approach is based on the idea that, if the observed negatively sloped delta functions for the Simon task are not specific, but merely result from the temporal distance between relevant and irrelevant response activations [16], then it should also be possible to produce such functions with the Eriksen flanker task. As we will show, this is indeed the case. Moreover, to examine details of the involved mechanisms, we fitted the DMC and an alternative drift-diffusion model to the corresponding flanker-task data.

Together, the results provide valuable insight into mental control processes involved in conflict paradigms. Before the results are reported, though, we briefly introduce the applied tasks and relevant concepts.

### Simon task

In the Simon task [5], a spatial response (e.g., pressing a ‘left’ or ‘right’ button) is required, depending on the specific (non-spatial) feature value (e.g., ‘red’ or ‘green’) of the stimulus (for an overview, see [6]). Moreover, the stimulus is presented to either the left or right of fixation. A typical result is that, although stimulus position is irrelevant, responses are usually faster and more reliable if the stimulus appears ipsilateral to the required response, compared to the contralateral position. This congruency effect is also called *Simon effect*.

A prominent account of the Simon effect is the dual-route model [11, 17]. It states that task-relevant stimulus information is transmitted via an *indirect* or *conditional* route, and that this information activates a response according to the required stimulus-response mapping. Additionally, though, a peripheral stimulus can activate the spatially corresponding response, which is presumably due to an automatic tendency to respond toward the source of stimulation. Because such response priming occurs even though stimulus location is task irrelevant, it is assumed that it proceeds automatically via a *direct* or *unconditional* route. The basic Simon effect is then explained by the assumption that the automatic activation through the direct route has a facilitating or interfering effect on response selection, depending on whether stimulus and response locations coincide or are opposite.

As mentioned, a characteristic property of the Simon effect in the latencies is that it decreases with RT (e.g., [14, 18, 19]). Accordingly, the delta functions are negatively sloped. Hommel [16, 20] has shown that automatically induced location-based response activation decays passively, which could be one reason for this phenomenon. However, the Simon effect can even be negative, which suggests that also other mechanisms affect the size of the Simon effect. De Jong [11], for instance, proposed that irrelevant response activation can be inhibited. Based on this idea, Ridderinkhof [10, 21] developed his *activation-suppression* hypothesis, which states that location-based response activation is actively suppressed to prevent unwanted responses to location. Because the suppression builds up gradually in time, it is more effective for slow than for fast responses, and can even lead to negative congruency effects. Meanwhile, a number of studies have examined different factors that produce modulations of the Simon effect (cf. [7]). Their results clearly show that the slope of the delta function strongly depends on the respective control demands in an experiment.

### Eriksen flanker task

In the Flanker task [3], a central target item is presented along with two or more irrelevant flanker items, which can be congruent or incongruent (for an overview, see [22]). For example, if participants have to respond to the letters ‘H’ and ‘S’ with a left and right button press, respectively, then ‘HHH’ and ‘SSS’ are congruent stimuli, whereas ‘SHS’ and ‘HSH’ are incongruent ones. In the latencies, the corresponding congruency effect usually increases with RT. Consequently, the delta functions are positively sloped. In contrast to the latencies, the congruency effect in the error rates usually decreases with RT, which is common for all conflict tasks. For analyzing the error rates, one often considers the so-called *conditional accuracy functions* (CAFs), which show how accuracy varies as function of RT. For congruent stimuli, accuracy is usually rather high even for fast responses and remains constant across RTs. In contrast, for incongruent stimuli accuracy is relatively low for fast responses, but for slow responses approaches the same level as for congruent stimuli.

Based on these characteristics of the congruency effects, a dual-process model has been proposed for the flanker task by Gratton and his coworkers [12, 23] and formalized as *Dual-Stage Two-Phase* (DSTP) model by Hübner, Steinhauser [4]. The basic idea of this drift-diffusion model is that flankers are processed automatically, which always activates their associated response. Therefore, some control is needed for producing reliable performance. The DSTP model includes an early and a late stimulus selection process. The early process is a perceptual filter of relatively low selectivity, whereas late selection operates on a conceptual level and is more selective.

### The present study

In the present study, we tested to what extent the control mechanisms in the Eriksen flanker task differ from those in the Simon task. Obviously, in both tasks response selection is affected by automatic activation produced by task-irrelevant stimulus features. However, the irrelevant features largely differ between the tasks. In the Flanker task, relevant as well as irrelevant features are similar and non-accidental (letter identity). The flanker letters are also used as targets and are irrelevant only, because they occur at locations that are irrelevant for the task. Because of the defined target location, the impact of irrelevant information can be restricted by spatial attention. In the Simon task, stimulus location is generally irrelevant and interferes only, because of its correspondence to the required response locations (left, right). Moreover, in contrast to the flanker task, relevant and irrelevant features are not separated in space.

Which of these differences are responsible for the differences in the delta functions? We assumed that the difference in spatial separation between relevant and irrelevant information is less essential. Rather, we hypothesized that the different types of irrelevant information is crucial, because they largely affect the temporal distance between the relevant and irrelevant activations [11]. In the flanker task relevant and irrelevant information are similar and, therefore, are processed similarly fast so that the corresponding activations occur close together in time. In the Simon task, however, the irrelevant stimulus location is usually processed faster than the relevant stimulus features. Consequently, there is, if at all, only partial overlap in time between the resulting activations so that there is a reduced conflict. Due to passive decay, the conflict is further reduced for slow responses. Moreover, in case the decay is not sufficient for a reliable performance, early irrelevant information might also require activation suppression [10].

If the temporal distance between relevant and irrelevant activation is indeed the essential difference between the tasks, then it should also be possible to produce negatively sloped delta functions with the Eriksen flanker task. This was tested in the present study. Our idea was to modulate the temporal overlap between relevant and irrelevant response activation by varying the stimulus-onset asynchrony (SOA) between flankers and target. By presenting flankers ahead of the target, irrelevant response activation should have more time to decay, or to trigger activation suppression before the relevant target information activates the correct response. We assumed that at least for some SOAs the flanker task should produce similar data as the Simon task, i.e. a reduced or even negative congruency effect for slower responses.

Varying the SOA in the flanker task has been done before [24–26]. However, most researchers did not analyze RT distributions. Merely Mattler [27] investigated the effect of SOAs on the delta functions for the flanker task. Although he found negative slopes when the flankers appeared before the target, they were rather small, and it was not tested whether they significantly deviated from zero. In the present study, we tried to produce a larger variation of delta functions. Ideally, they should not only be negatively sloped, but also indicate a negative congruency effect, at least for longer SOAs. As we will see, this goal was achieved in our second experiment.

To get some insight into possibly involved control mechanisms, we also fitted the mentioned drift-diffusion models to the data. Furthermore, for demonstrating that our SOA flanker-task data are indeed similar to Simon-task data, we also conducted a Simon-task experiment (Experiment 3). Finally, we show that the models fitted to the flanker-task data also account for the Simon-task data.

## Experiment 1

The aim of our first experiment was to vary the temporal separation of relevant and irrelevant response activation in an Eriksen flanker task. For this objective, flankers were presented 17, 100, or 400 *ms* before the target. With this selection, we had a delayed target onset in all conditions. However, for the 17-*ms* SOA we expected similar congruency effects as for a simultaneous presentation of target and flankers. The congruency effect was expected to be larger for the 100-*ms* SOA condition, because the flankers have more time for interference, compared to the 17-*ms* SOA [25, 28]. In contrast, in the 400-*ms* SOA condition the irrelevant activation should have been largely decayed. Accordingly, the congruency effect should be relatively small, as in Mattler [27].

The SOA variations were expected to produce specific effects on the delta functions. For the 17-*ms* SOA, the delta function should increase, as is usually observed in the flanker task. In contrast, for the two longer SOAs, we expected delta functions similar to those usually observed for the Simon task [10], i.e. the slopes of the functions should be negative. This would also be in line with the results of Mattler [27]. Finally, we thought that it was possible, at least for the 400-*ms* SOA, that a negative congruency effect might occur for slow responses, which would signal activation suppression [10].

Besides the mean results and delta functions, we will also report CAFs, because they show how the congruency effect in the error rates varies with RT. This provides further information about the involved mental control processes.

### Method

Participants. Sixteen students (5 men; 19 to 36 years; mean age: 22.2 years) from the Universität Konstanz were recruited via an online-system ORSEE [29] to participate in the experiment and were paid (8€/*hr*) or received course credit. During the registration process in our online recruitment system, the participants have been informed that they can withdraw from any study at any time without further consequences and that the data will be used for scientific publication. Registration could be completed only when the participants have accepted these rules by setting a corresponding check mark. Given this general acceptance, only a verbal informed consent statement was obtained prior to participation in this specific study. It was documented on a sheet together with the received compensation. The study complied with national and local guidelines and was approved by the Institutional Review Board of the Universität Konstanz.

#### Apparatus

The stimuli were presented on a 19”-monitor with a resolution of 1280×1024 pixels. A personal computer (DELL, Intel Pentium Dual-Core E2200) served for controlling stimulus presentation and response registration. The software Presentation (Version 14.7 Build 11.10.10, www.neurobs.com) was used for programming and running the experiment.

#### Stimuli

The stimulus set consisted of the letters ‘H’ and ‘S’. The height of the letters subtended a visual angle of 1.27° at an approximate viewing distance of 45 *cm*. The average width of the letters was 0.89°. Items were presented in white on a black background and the target always appeared at the central position of the screen. Flankers consisted of two copies of a letter, which were presented left and right of the target at an eccentricity of 1.27°.

#### Procedure

**Fig 1:**
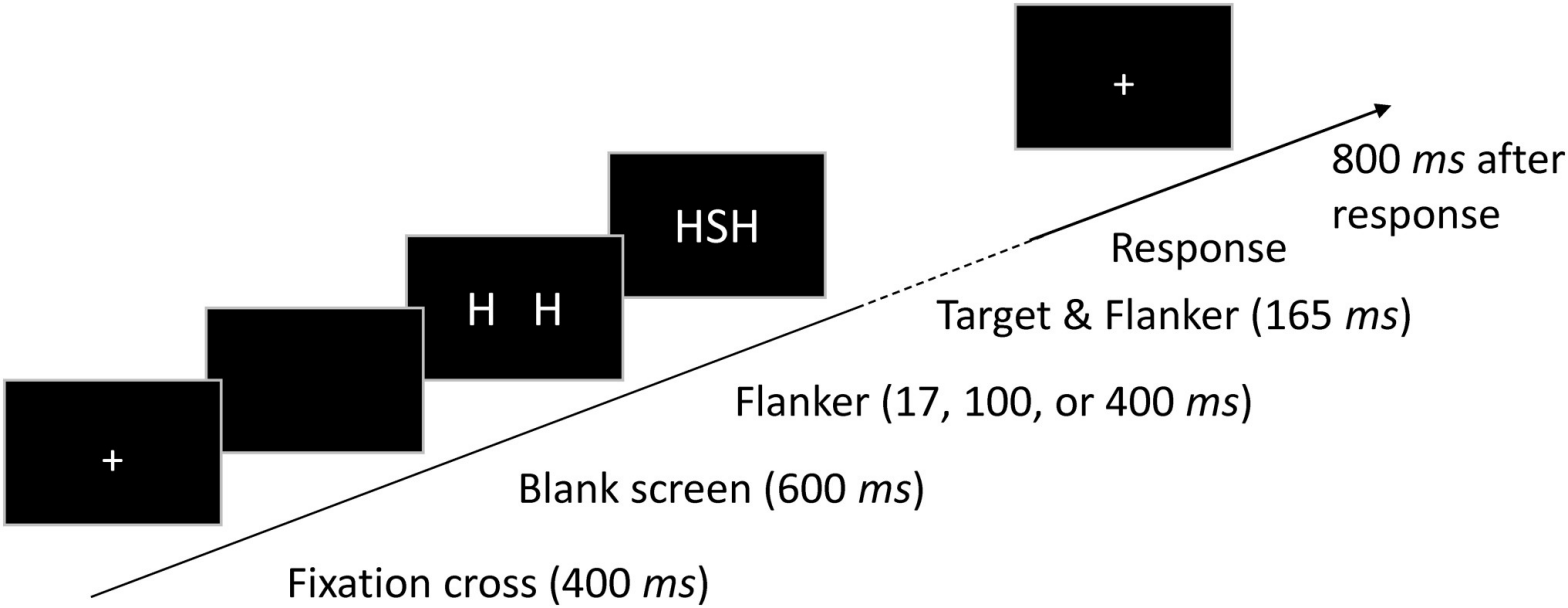
Procedure of Experiment 1.

Each trial started with the presentation of a fixation cross for 400 *ms*. After a blank screen for 600 *ms*, the flankers appeared, followed by the target after a randomized SOA of 17 (precisely, 1000/60), 100, or 400 *ms*. The SOAs were similar to those used by Mattler [27]. However, instead of 0 *ms* we chose 17 *ms*, because we thought that a simultaneous presentation of target and flanker might also be qualitatively different from the two longer SOAs. Target and flankers remained visible together for 165 *ms*. It should be noted that with this procedure flanker duration increased with an increasing SOA. Participants had to indicate the target letter ‘H’ or ‘S’ by pressing a left or right key with the index or middle finger of their right hand, respectively. Half of the stimuli were congruent, the other half were incongruent. 800 *ms* after the response, the next trial began (see Fig 1). Errors were signaled by a short beep.

Participants were instructed to respond as fast and as accurate as possible. Moreover, if they made more than 15% errors within one block, they were instructed to respond more accurately. After 32 practice trials, the participants worked through 14 test blocks of 96 trials, each. Altogether, the session lasted 70 minutes.

### Results

Data were analyzed and visualized with R [30]. Responses faster than 100 *ms* or slower than 1500 *ms* were excluded from analysis (< 0.4% of the data).

#### Response times

Mean response time of correct responses was 437 *ms* (*SD* = 69.7). Latencies of correct responses were subjected to a within-participant ANOVA with the factors *congruency* (congruent, or incongruent), and *SOA* (17, 100, or 400 *ms*). The main effect of *congruency* was significant, *F*(1, 15) = 60.8, *p* < 0.001, η_p_^2^ = .802. Responses to congruent stimuli were faster than to incongruent ones [420 *ms* (*SD* = 69.1) vs. 454 *ms* (*SD* = 66.9)]. The factor *SOA* was also significant, *F*(2, 30) = 143, *p* < 0.001, indicating that RT decreased with an increasing SOA [473 *ms* (*SD* = 67.2), 438 *ms* (*SD* = 62.8), and 400 *ms* (*SD* = 60.7)]. However, there was also a reliable interaction between the two factors, *F*(2, 30) = 15.5, *p* < 0.001, η_p_^2^ = .508. The congruency effect was largest (Δ51 *ms*) for the 100-*ms* SOA, smallest (Δ10 *ms*) for the 400-*ms* SOA, and in-between (Δ39 *ms*) for the 17-*ms* SOA.

#### Error rates

Mean error rate was 7.95% (*SD* = 3.71). An ANOVA of the same type as for the RTs was also conducted for the error rates. The factor *congruency* was significant, *F*(1, 15) = 15.1, *p <* 0.01, η_p_^2^ = .501, indicating that participants made more errors for incongruent than for congruent stimuli [9.48% (*SD* = 3.18) versus 6.43% (*SD* = 3.60)]. There was also a significant interaction between *SOA* and *congruency*, *F*(2, 30) = 13.4, *p* < 0.001, η_p_^2^ = .471. The congruency effect was largest (Δ5.49%) for the 100*-ms* SOA, smallest (Δ0.746%) for the 400*-ms* SOA, and in-between (Δ4.39%) for the 17*-ms* SOA.

#### Distributional analyses

**Fig 2:**
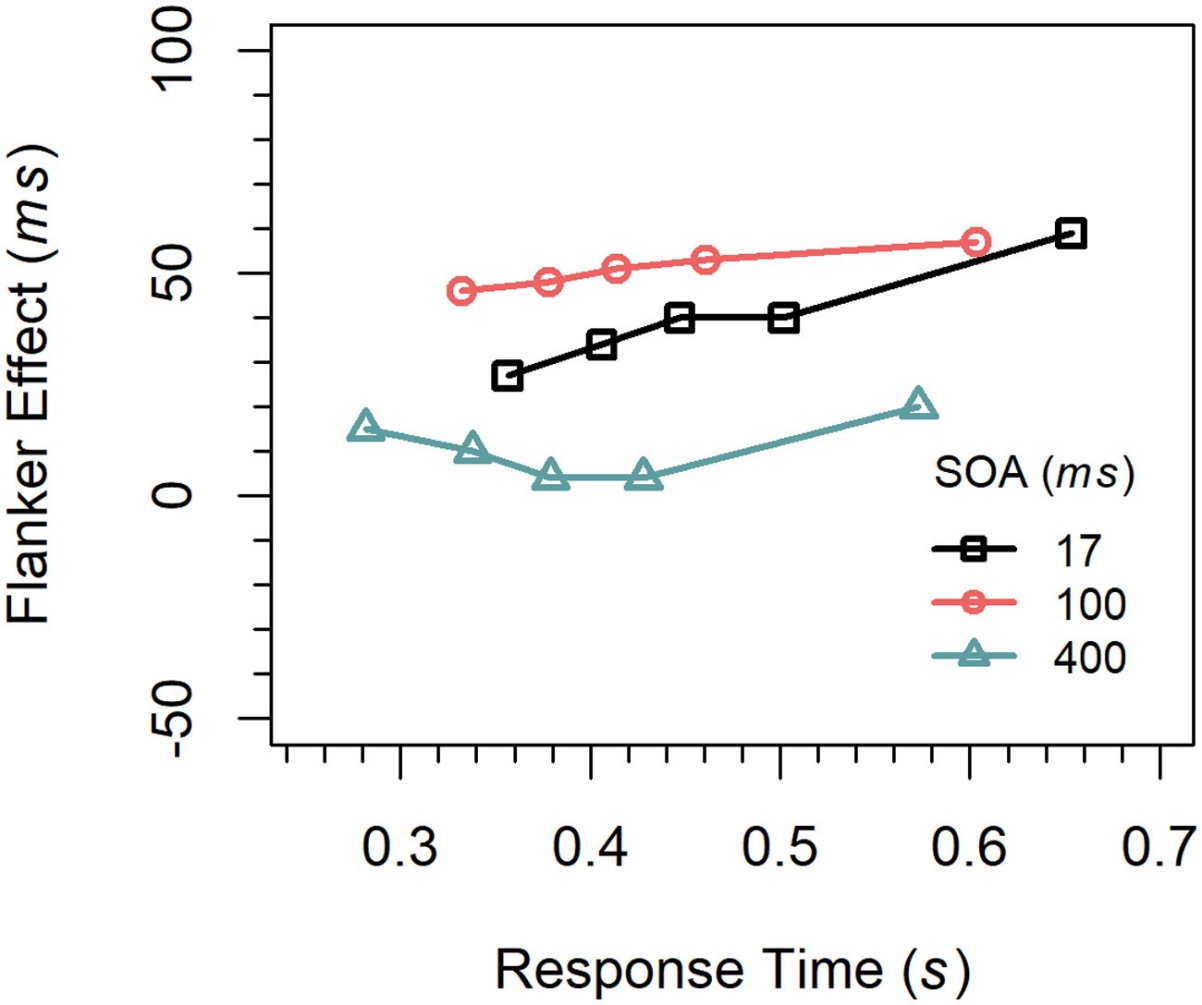
Delta functions for the different conditions in Experiment 1.

Delta functions. We first computed cumulative distribution functions for the latencies of correct responses for each congruency condition (congruent, incongruent) and SOA (17, 100, 400 *ms*) by quantile-averaging (.1, .3, .5, .7, .9) the corresponding data [31]. The delta function for a given SOA was then calculated by subtracting the quantile RTs for congruent trials from those for incongruent trials, respectively, and relating the results to the corresponding mean quantile RTs. The results are shown in Fig 2. As can be seen, the delta function was increasing for the 17-*ms* SOA, almost flat for the 100-*ms* SOA, and partly decreasing in the 400-*ms* SOA.

To test whether the slopes differ reliably, the data were entered into a three-factor ANOVA for repeated measurements with the factors *quantile*, *SOA*, and *congruency*. Changes in the slope of the delta functions would be reflected by an interaction of the factors quantile and congruency. Therefore, only interactions involving these two factors are reported. There was a significant two-way interaction between the factors *congruency* and *quantile*, *F*(4,60) = 4.59, *p* < 0.01, η_p_^2^ = .234, indicating that the congruency effect increased with RT (29, 30, 31, 33, 46 *ms*), i.e. that there was a general positive slope in the delta functions. The three-way interaction was not significant, indicating that the slopes did not differ between the delta functions, *F*(8, 120) = 1.06, *p =* 0.364, η_p_^2^ = .069. Because this method might not be optimal for testing the slopes, we also computed the slopes directly by linear regression for each participant and SOA. For the 17-*ms* SOA the mean slope of 0.548 (standardized) was significantly greater than zero, *t*(15) = 2.97, *p* < 0.01, Cohen’s *d* = 0.742. For the two longer SOAs the slopes (0.125, 0.035) were numerically also positive, but did not significantly differ from zero (*p*s > 0.5). Power analyses revealed that in order for effects of this size (*d* = 0.160, *d* = 0.044) to be detected (80% chance) as significant at the 5% level (one-sided), samples of 243 and 3246 participants would be required, respectively.

**Fig 3:**
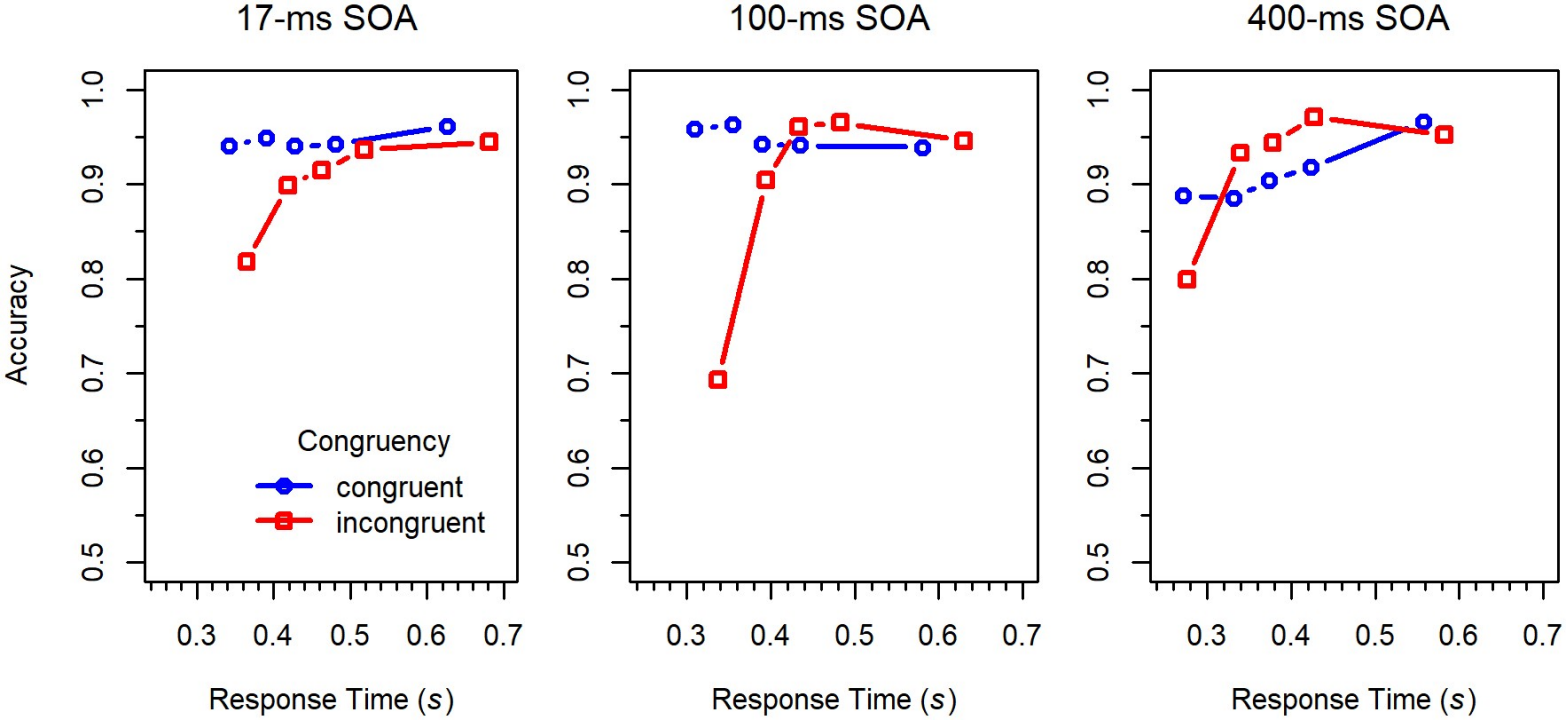
Conditional accuracy functions for the different conditions in Experiment 1.

Conditional accuracy functions. CAFs were computed by sorting the RT data of each participant and condition into five 20% bins, and by calculating mean RT and proportion of correct responses for each bin. The obtained values were then averaged across participants [10]. As can be seen in Fig 3, accuracy was lowest for the fastest responses to incongruent stimuli, which was especially pronounced for the 100-*ms* SOA condition. However, accuracy increased with RT, and for the shortest two SOAs, quickly approached the same high level as the accuracy for congruent stimuli. For the 400-*ms* SOA, accuracy for congruent stimuli was relatively low for fast responses, compared to the other SOAs. This even produced a negative Simon effect in the medium RT range.

### Discussion

As in former SOA studies with the flanker task [25, 28], the congruency effect largely varied with the delay between flankers and target. It was largest for the 100-*ms* SOA, and smallest for the 400-*ms* SOA. The small effect in the 400-*ms* SOA condition suggests that the irrelevant activation produced by the flankers had largely decayed, or already been suppressed when the target arrived.

The CAFs for the 17-*ms* SOA are similar to those of the standard flanker task (e.g., [32]). Compared to this condition, the error rate for the 100-*ms* SOA was substantially increased for fast responses to incongruent stimuli. Such an increase was not observed for the 400-*ms* SOA. However, in that condition, congruent stimuli produced more errors than incongruent ones in the medium range of RTs, which is difficult to explain by a passive decay of irrelevant activation [16, 20]. Thus, it seems that there was also some kind of activation suppression in the 400-*ms* SOA condition [10, 21].

Concerning the delta functions, inspection of Fig 2 reveals that they differ more in their vertical position than in slope. Although all slopes were numerically positive, only that for the 17-*ms* SOA was significantly greater than zero. The slopes of the other functions decreased slightly with increasing SOA, but not enough to be negative, which is similar to the results of Mattler (27)]. These results demonstrate that a temporal distance between relevant and irrelevant activation in the flanker task is not sufficient for producing negatively sloped delta functions as usually observed for the Simon task.

Do our results imply that the temporal-distance hypothesis is wrong? After reconsidering the details of our experiment, we hypothesized that negatively sloped delta functions might only be observed if response activation from an irrelevant source is suppressed. Because suppression is favorable only for incongruent stimuli, but disadvantageous for congruent ones, suppression speeds up responding on incongruent trials, but slows down responding on congruent ones, which can even lead to a reversed congruency effect. Thus, an optimal strategy would be to suppress irrelevant activation only for incongruent stimuli. However, for such a *conditional* activation suppression it would be necessary to identify quickly the congruency type of the stimulus. In the present case, this could indeed have been possible, because congruent stimuli always consisted of identical letters, whereas incongruent ones included different letters. If the equality of letters was detectable rather quickly, then suppression could have been stopped or even prevented. That activation suppression can strategically be adapted to task demands has already been shown for the Simon task (e.g., [7]) as well as for the flanker task (e.g., [26]). To test whether our reasoning is valid, we conducted an experiment, where for some stimuli it was not easy to detect whether the stimulus is congruent or not.

## Experiment 2

Our second experiment was similar to the first one, except that four stimuli (letters) were mapped onto the two responses. Accordingly, for half of the congruent stimuli the flankers were again equal to the target (congruent-same), whereas for the other half they were different (congruent-different). Consequently, if the target letter appeared and was identical to the flanker, then it could still be concluded that the stimulus was congruent. However, if the target letter was different, then the stimulus was incongruent only in two third of the cases. Thus, a more complex control strategy was required in this experiment.

We expected that response suppression should again be low when the letters for target and flankers are identical. However, when they are different, then irrelevant response activation should strongly be suppressed, which should produce large negative effects for congruent-different stimuli. Consequently, the delta functions should largely differ between the two congruent stimulus types. Specifically, for congruent-different stimuli the slopes for the longer SOAs should now be negative.

### Method

Eighteen participants (6 men; age: 18–26 years, mean: 22.1 years) were recruited at the Universität Konstanz. All had normal or corrected to normal vision and were paid (8€/*hr*) or received course credit for their participation. Apparatus and procedure were the same as in Experiment 1, except that four (‘H’, ‘K’, ‘B’, and ‘S’) instead of two letters were used as item set. ‘H’ and ‘K’ were mapped to the right response button, whereas ‘B’ and ‘S’ were mapped to the left button. All letter pairs occurred equally often. Consequently, incongruent, congruent-same, and congruent-different stimuli occurred on 50, 25, and 25% of the trials, respectively.

### Results

Responses faster than 100 *ms* and slower than 1500 *ms* were excluded from analysis (< 0.5% of the data).

#### Response times

Mean response time of correct responses was 476 *ms* (*SD* = 65.9). Latencies of correct responses were subjected to an ANOVA for repeated measures with the factors *congruency* (congruent-same, congruent-different, or incongruent), and *SOA* (17, 100, or 400 *ms*). The main effect of *congruency* was significant, *F*(2, 34) = 46.5, *p* < 0.001, η_p_^2^ = .732, indicating that responses to congruent-same stimuli were faster (459 *ms*, *SD* = 68.3) than those to congruent-different (480 *ms*, *SD* = 64.2) and incongruent stimuli (489 *ms*, *SD* = 62.5). The main effect of *SOA* was also reliable, *F*(2,34) = 100, *p* < 0.001, η_p_^2^ = .855. RTs decreased with an increasing SOA [504 *ms* (*SD* = 64.6), 471 *ms* (*SD* = 64.0), and 453 *ms* (*SD* = 59.6)]. However, these effects were qualified by a significant interaction between the two factors, *F*(4, 68) = 6.94, *p* < 0.001, η_p_^2^ = .290. For the 17-*ms* condition, the latencies for the congruency conditions (congruent-same, congruent-different, and incongruent) were 494 *ms* (*SD* = 68.9), 501 *ms* (*SD* = 63.5), and 519 *ms* (*SD* = 62.2), for the 100-*ms* condition 449 *ms* (*SD* = 64.9), 473 *ms* (*SD* = 65.6), and 490 *ms* (*SD* = 57.5), and for the 400-*ms* condition 434 *ms* (*SD* = 59.4), 467 *ms* (*SD* = 61.7), and 459 *ms* (*SD* = 55.8), respectively. If we consider the individual RTs, then we see that the difference between congruent-different and congruent-same was very small in 17-*ms* SOA condition, medium in 100-*ms* SOA condition, and largest in the 400-*ms* SOA condition. Interestingly, in the latter condition RTs for congruent-different stimuli were even longer than those for incongruent stimuli.

#### Error rates

The mean error rate was 7.04% (*SD* = 4.05). An ANOVA of the same type as for the RTs was also conducted for the error rates. It revealed a significant main effect of *congruency*, *F*(2, 34) = 14.7, *p <* 0.01, η_p_^2^ = .464, indicating fewer errors for congruent-same (5.51%, *SD* = 3.09) than for congruent-different (7.65%, *SD* = 4.40) and for incongruent stimuli (7.98%, *SD* = 4.16). However, there was also a significant interaction between *congruency* and *SOA*, *F*(4, 68) = 10.2, *p* < 0.001, η_p_^2^ = .376. For the 17-*ms* SOA condition, the error rates for the different congruency conditions (congruent-same, congruent-different, and incongruent) were 5.88% (*SD* = 2.47), 5.98% (*SD* = 3.95), and 9.35*%* (*SD* = 5.00). For the 100-*ms* SOA condition the error rates were 5.67, 6.72, and 9.00%, and for the 400-*ms* SOA condition they were 4.98% (*SD* = 3.06), 10.2% (*SD* = 5.16), and 5.58% (*SD* = 2.37). Thus, the congruency effects were positive in the 17-*ms* and the 100-*ms* SOA conditions, but negative between congruent-different in incongruent in the 400-*ms* SOA condition. A further analysis revealed that this negative effect was reliable, *F*(1,17) = 26.5, *p* < 0.001, η_p_^2^ = .609.

#### Distributional analyses

**Fig 4:**
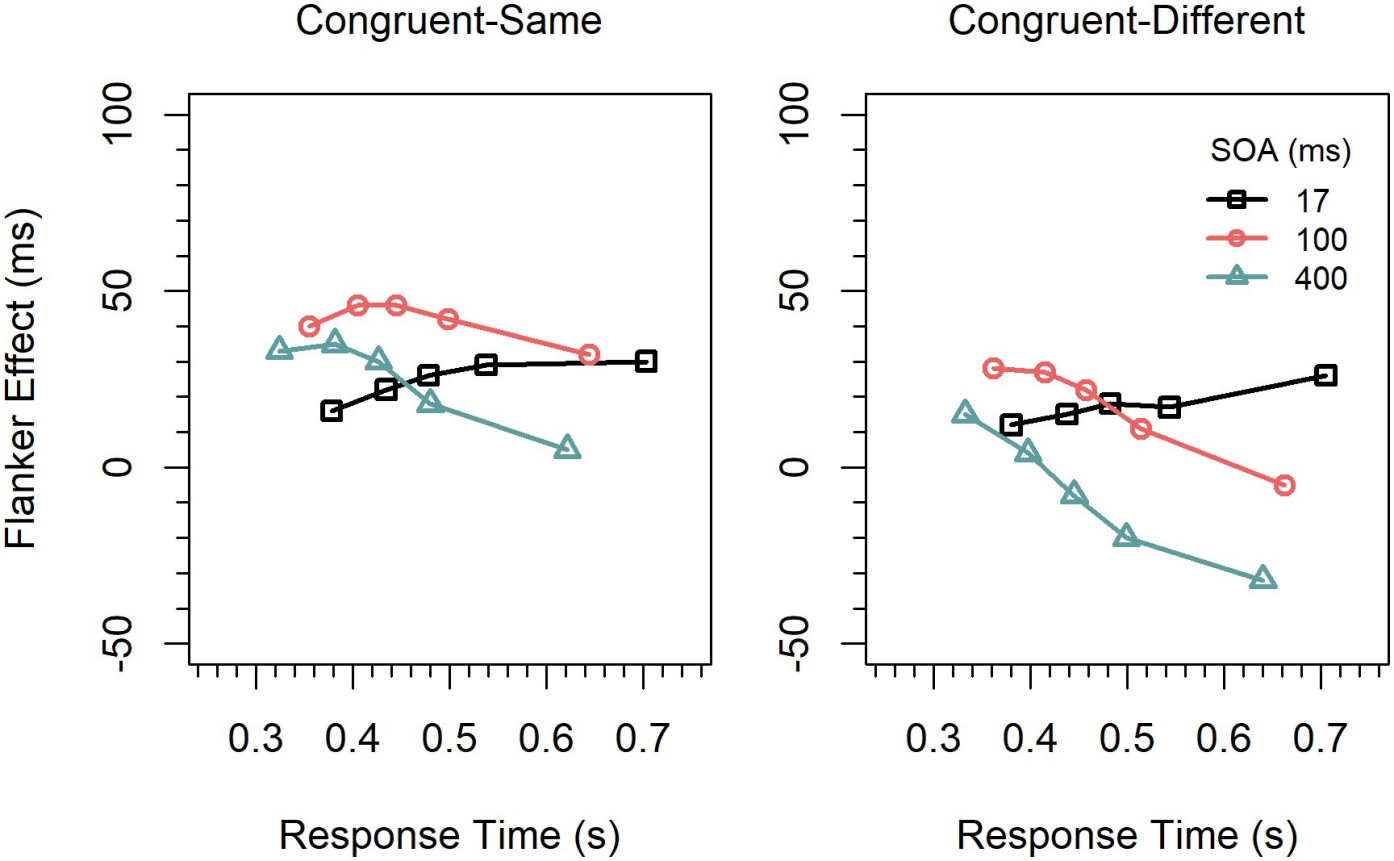
Delta functions for the different conditions and stimulus types in Experiment 2.

Delta functions. Delta functions for the three SOA conditions were computed separately for congruent-same and congruent-different stimuli. The resulting functions for the two stimulus types are shown in Fig 4. They were first analyzed by an ANOVA similarly as in Experiment 1, except that we added the factor *condition* (congruent-same, or congruent-different). An overall analysis revealed a significant two-way interaction between the factors *quantile* and *condition*, *F*(8,136) = 4.67, *p* < 0.001, η_p_^2^ = .215, which, however, was qualified by a reliable three-way interaction between *quantile*, *condition*, and *SOA*, *F*(16, 272) = 4.00, *p* < 0.001, η_p_^2^ = .190.

To analyze to what extent this interaction was driven by differences between the slopes, we computed the slopes by linear regression for each participant, SOA, and stimulus condition (congruency-same, congruency-different). For the congruent-same condition, the slope for the 17-*ms* SOA was significantly greater (0.444) than zero, *t*(17) = 2.89, *p* < 0.01, Cohen’s *d* = 0.681, whereas that for the 400-*ms* SOA was significantly smaller (-0.507) than zero, *t*(17) = -3.63, *p* < 0.01, *d* = -0.856. The slope (-0.170) for the 100-*ms* SOAs did not significantly differ from zero, *t*(17) = -0.948, *p* = 0.356, *d* = -0.223. Power analysis revealed that in order for an effect of this size to be detected (80% chance) as significant at the 5% level (one-sided), a samples of 125 participants would be required.

For the congruent-different condition, the slope (0.282) for the 17-*ms* SOA shortly failed to reach significance, *t*(17) = 1.72, *p* = 0.052, *d* = 0.406. Power analysis revealed that in order for an effect of this size to be detected (80% chance) as significant at the 5% level (one-sided), a samples of 39 participants would be required. The slopes (-0.544, -0.739) for the two other SOAs were significantly smaller than zero; *t*(17) = -4.17, *p* < 0.001, *d* = -0.982; *t*(17) = -7.18, *p* < 0.001, *d* = -1.69. Because the slopes for the 400-*ms* SOA were significant in both conditions, we also tested their difference and found that the slope in the congruent-different condition was significantly more negative than that in the congruent-same condition, *t*(17) = 2.40, *p* < 0.05, *d* = 0.565.

**Fig 5:**
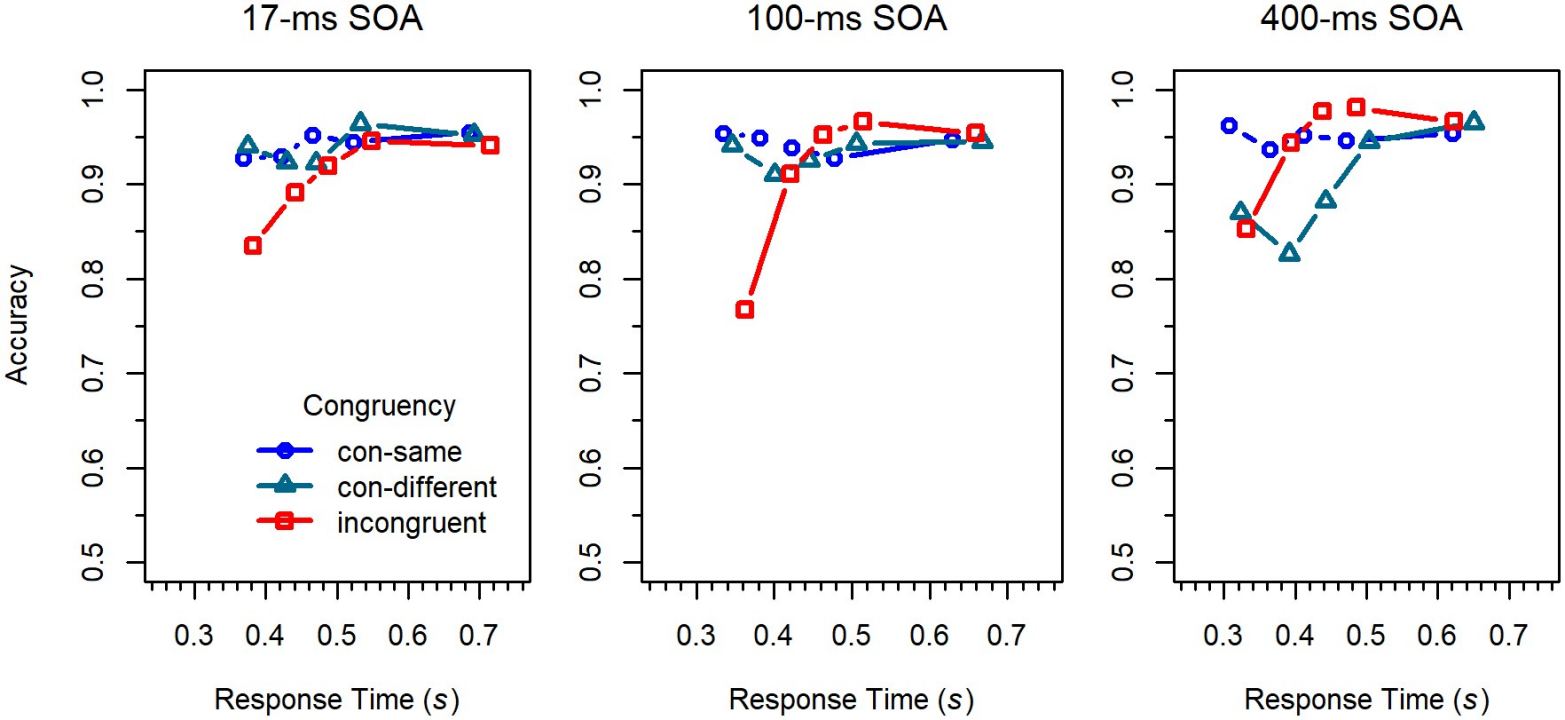
Conditional accuracy functions for the different conditions in Experiment 2.

Conditional accuracy functions. The CAFs for the three congruency conditions are shown in Fig 5. As can be seen, the pattern was, by and large, similar to that in the previous experiment. However, the reversal of the Simon effect in the medium range of RTs for the 400-*ms* SOA conditions was much more pronounced, especially for the congruent-different condition. Moreover, whereas there was hardly any difference between congruent-same and congruent-different flankers in the 17-*ms* and the 100-*ms* SOA conditions, it was rather extreme in the 400-*ms* SOA condition.

### Discussion

The overall pattern of effects was similar to that in the previous experiment, i.e., the effect was largest for the 100-*ms* SOA, medium for the 17-*ms* SOA, and smallest for the 400-*ms* SOA. This time, however, there were also negatively sloped delta functions. Moreover, the slopes differed not only between the SOAs, but also showed a different pattern in the two stimulus conditions (Fig 4). In the congruent-same condition the slope was again positive for the shortest SOA, as in the previous experiment. However, different from that experiment, the slope for the 100-*ms* SOA was now negative. In the congruent-different condition, the slope for the 17-*ms* SOA was not significantly different from zero, but those for the two longer SOAs were significantly negative. Moreover, the delta function for the 400-*ms* SOA was significantly more negatively sloped than the corresponding function in the congruent-same condition. Thus, with the different set of stimuli applied in this experiment we were obviously successful in inducing response-activation suppression. That there was indeed suppression is further supported by the result that the flanker effect was negative for the slower responses in the 400-*ms* SOA condition. In the error rates for this condition, there was also a reversed flanker effect across a wide medium range of RTs, which was especially pronounced for congruent-different stimuli, as can be seen in Fig 5.

These results confirm our hypothesis that the participants in Experiment 1 quickly detected whether the stimulus was congruent or not, and adjusted the strength of suppression accordingly. This was possible, however, only because congruent flanker letters were always identical to the target letter. In the present experiment, where congruent flanker letters could also be different from the target letter (congruent-different), the participants were not generally able to detect congruent stimuli, but only those with identical letters (congruent-same). For the latter stimuli, they again used a reduced activation suppression.

A possible account of our results could be as follows: After flanker onset, the participants started suppressing the automatically emerging response activation to some extent. When the target appeared, they quickly assessed whether the letter was identical to the flankers or not. If so, suppression was ended or reduced; if not, suppression was continued and its strength eventually increased. This strategy was beneficial for responding to congruent-same and incongruent stimuli. For responding to congruent-different stimuli, though, it was harmful, because in this case the correct response was inhibited. This inhibition produced a performance that could even be worse than that for incongruent stimuli, especially for long SOAs, which explains that there were also negative congruency effects. The result that there was also some suppression in the congruent-same condition, at least for the 400-*ms* SOA, which is different from Experiment 1, can be explained by the fact that this condition only occurred on 25% of trials, compared to 50% in the previous experiment. Due to the smaller proportion the basic level of suppression was presumably generally increased relative to Experiment 1.

Taken together, the results of this experiment not only show that negatively sloped delta functions can also be observed for the flanker task, given appropriate conditions, but also that participants are in principle able to adjust activation suppression on-the-fly to meet current control demands. In order to gain deeper insight into the involved control mechanisms, we modeled the data of this experiment. The applied models will be introduced next.

### Modeling

To examine possible processes involved in our SOA flanker task, we fitted the DMC model [15] and the DSTP model [4] to the data of Experiment 2. We did not consider the shrinking spotlight model [33], which can also account for standard flanker-task performance, because its structure is less flexible, and, therefore, seemed to be inappropriate for modeling our specific data.

The DMC model has been developed as general model for performance in conflict tasks, and was already applied to the flanker task and the Simon task. The DSTP model, up to now, has only been fitted to data from the standard flanker task [4, 32, 34], and to data from the global/local task [35]. Both of these tasks produce congruency effects that increase with RT, i.e., positively sloped delta functions. Some authors have claimed that the DSTP model cannot produce negatively sloped delta functions (e.g., [34, 36]). However, this only holds for its standard interpretation. Here, we show that the DSTP framework is rather flexible.

As we have seen, the mean difference in Experiment 2 between congruent-same and congruent-different was practically absent (7 *ms*) for the 17-*ms* SOA condition, and negligible (24 *ms*) for the 100-*ms* SOA condition. Therefore, for simplicity, the data for the two congruent stimulus types were collapsed for these two SOA conditions, so that we only had two conditions for modeling: congruent and incongruent. The data from the 17-*ms* SOA condition are considered as representative for those of the standard flanker task. Accordingly, the standard interpretation of the DSTP model was applied. Fitting the data of the 100-*ms* SOA condition was a challenge, because in this case the delta functions were negative. Thus, we had to interpret the DSTP model differently. Finally, for the 400-*ms* SOA condition, all three congruency conditions were modeled.

Because the DSTP model as well as the DMC model are variants of the drift-diffusion model, we will briefly consider its basic mechanisms.

#### The diffusion process

Both the DSTP and the DMC model are based on a response-selection mechanism, implemented as diffusion process (cf. [37]). Such a process is characterized by a drift rate *μ* reflecting the evidence available for response A relative to response B and by two corresponding thresholds *A* and–*B*. Responses A and B usually represent a correct and a wrong response, respectively. Noisy samples of the evidence are accumulated beginning at *t*_*0*_ with state *X*_*start*_, until threshold *A* or–*B* is reached. The duration of this process is the decision time. It is assumed that the response time is the sum of this decision time and some non-decisional time *t*_*nd*_, representing the duration of stimulus encoding and response execution. The complexity of the diffusion process can further be increased by assuming that the starting state, the non-decisional time, and/or the rate vary randomly across trials according to specific distributions [38].

Apart from the assumption of a single response-selection process, the DMC and DSTP models differ largely in their architecture. For instance, whereas the drift rate for response selection changes gradually during response selection in the DMC model, it is assumed to change abruptly in the DSTP model. Currently, it is disputed which assumption is more appropriate [32, 33, 35].

In the following, we will describe the two models in detail and report the corresponding fit methods and results. For both models, we assume that evidence accumulation starts with target onset, which is compatible with measuring the RTs. Because the information provided by the flankers is uninformative with respect to the required response, its accumulation would make no sense. However, this does not mean that flankers presented ahead of the target have no effect. They clearly activate their associated response. Usually, it is assumed that the rate of evidence accumulation is the difference in activation between the two response alternatives. A pre-activation by the flankers would then bias the rate in favor of the response associated with the flankers. Consistent with this reasoning, the starting point *X*_*start*_ (or its mean) was set to zero for all SOA conditions, whereas the initial rate of evidence accumulation was assumed to vary with the available pre-target information.

### The DMC model

The specific idea for the DMC model [15] is similar to that for the dual-route model [11, 17]. It is assumed that task-relevant and task-irrelevant activations result from a controlled and from an automatic process, respectively, and that these activations are transmitted through separate, parallel processing pathways. Moreover, whereas the rate *μ*_*c*_ representing the controlled process remains constant, the rate *μ*_*a*_(*t*) resulting from the automatic processes varies as a function of time *t*. The dynamics of this rate is modeled by a Gamma density function with shape parameter *a* > 1 and scale parameter *τ*. The function is further scaled (multiplied) by a parameter *m* reflecting the strength of automatic activation. If one integrates the time function *μ*_*a*_(*t*) to inspect the expected evidence provided by the automatic process at time *t*, then the resulting *accumulated-rate* function resembles an impulse function (see Figure B1 in [15]). The fact that this function (the accumulated rate) finally approaches zero, implies that a positive rate at the beginning has to be counterbalanced by a negative rate in the further course, or vice versa. For Ulrich, Schröter [15] the impulse function represents automatic activation. However, we think that the rate as such rather than their sum better reflects automatic activation. With this interpretation a phase of automatic activation is always followed by a phase of inhibition.

The DMC model further assumes that the relevant and irrelevant activations superimpose. This means that the overall drift rate at time *t* is the sum of the rate for the controlled process and the rate at time *t* for the automatic process. Furthermore, it is assumed that the rate of the automatic process is of the same size for congruent and incongruent stimuli, but that its sign is negative for incongruent stimuli, which can easily be achieved by scaling the rate with–*m* instead of *m*. Thus, the overall rate for selecting a response to a congruent stimulus is *μ*(*t*) = *mμ*_*a*_(*t*) + *μ*_*c*_. Additionally, it is assumed that *X*_*start*_ varies across trials according to a general beta distribution centered symmetrically around zero with standard deviation *σ*_*start*_, and that *t*_*nd*_ varies according to a normal distribution with mean zero and standard deviation *σ*_*tnd*_ (for details, see [15]). Altogether, the number of parameters for the DMC model adds up to eight.

#### Fit procedure

A computer-simulation version of the DMC model was fitted to the distributional data of Experiment 2. The procedure was similar to that in Hübner et al. [4] for the DSTP model. Specifically, the PRAXIS routine [39, 40], which is based on Powell’s [41] algorithm, was applied to find parameter values for a given model. For the 17-*ms* and 100-*ms* SOAs the congruent and incongruent conditions were fit together, which resulted in 22 bins for each SOA condition. For correct responses, there were six bins for each CDF, which resulted from the respective five quantiles (.1, .3, .5, .7, .9). Thus, there were six bins for the congruent condition, and six bins for the incongruent condition. Error proportions were extracted from the CAFs, i.e., from the corresponding individual five bins of the CAFs. Accordingly, there were five bins for errors in the congruent condition, and five bins for errors in the incongruent condition. The method was introduced by Hübner [35] and has recently positively been tested by White, Servant [36]. Its great advantage is that, in contrast to CDFs, CAFs can also be computed for conditions with few or no errors. Thus, there were 2 × 6 bins for the CDFs and 2 × 5 bins for the CAFs, summing up to 22 bins for each of the two shorter SOA condition.

For the 400-*ms* SOA condition, we had to handle three congruency conditions. Accordingly, just reversing the scaling parameter *m* was not possible. For fitting all three conditions together, we would have had to increase the number of parameters considerably. According to our experience, however, such a number cannot reliably be estimated by a fit procedure based on simulated data. Therefore, each congruency condition (11 bins; 6 for the CDF, 5 for the CAF) was fitted individually, which greatly reduced the degrees of freedom (*df*). Nevertheless, this approach was sufficient for examining how the parameters differ between the conditions.

Starting from different sets of parameter values to avoid local minima, each fit continued until the *G*^2^ (Wilks likelihood ratio chi-square) statistics, which approximates the χ^2^ statistics as sample sizes become large, was minimized. With this goodness-of-fit measure both the distribution shapes and the response probabilities are taken into account simultaneously, because it reflects how well the proportion of observations in each of the bins fits to that predicted by the model (cf. [42]). However, as Ratcliff and Smith [42], we have used the *G*^2^ statistic as a relative rather than absolute measure of fit. Each fit usually required several hundred iterations, in each of which 8·10^5^ trials were simulated.

#### Modeling results

**Fig 6:**
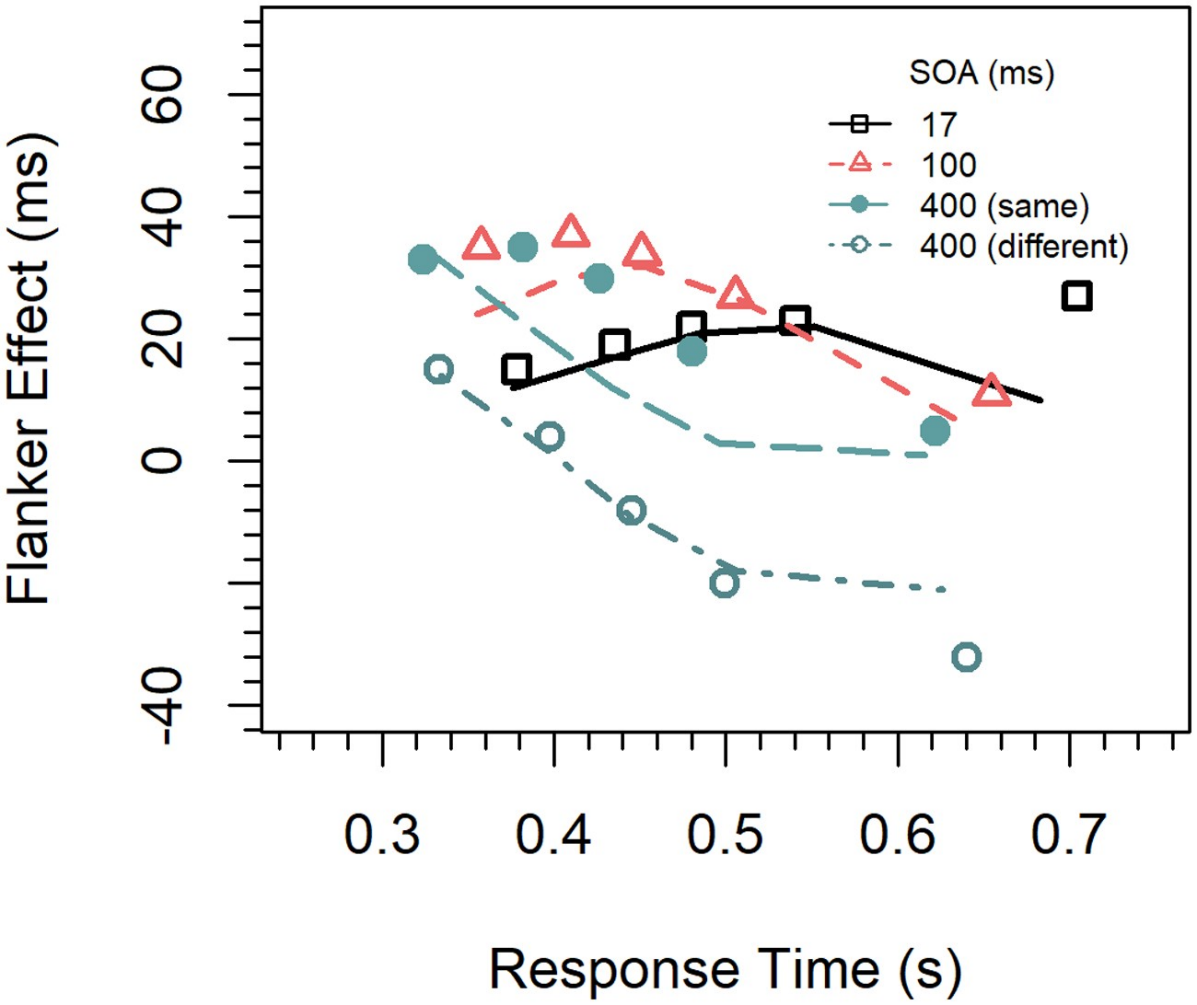
Delta functions for the different conditions in Experiment 2 (data points). The lines represent the fit of the DMC model.

**Fig 7:**
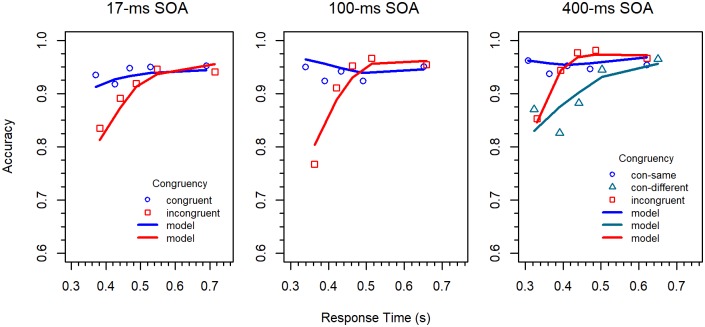
CAFs for the different conditions in Experiment 2 (data points). The lines represent the fit of the DMC model.

The fits of the model to the delta functions and the CAFs are shown in Figs 6 and 7, respectively. The corresponding parameters and goodness-of-fit measures are provided in Table 1. As can be seen, the fit to the delta functions is relatively good. Merely the congruency effect for slow responses in the 17-*ms* SOA condition is underestimated. This is due to a specific characteristic of the DMC model, which will be considered later. The obtained values for the CAFs are also close to the data. If we consider the parameters, then we see that *m* was somewhat larger for the 100-*ms* SOA condition than for the 17-*ms* condition, suggesting that automatic processes contributed more to the performance in this condition. However, the automatic processes were strongest, i.e. the magnitude of *m* was largest, for incongruent stimuli in the 400-*ms* SOA condition.

**Table 1:** Estimated parameters obtained by fitting the DMC model to the data of Experiment 2.

SOA (*ms*)	Parameters								*df*	*G*^*2*^	*BIC*
	*μ*_*c*_	*m*	*σ*_*start*_	*A*/*B*	*a*	*τ*	*σ*_*tnd*_	*t*_*nd*_			
17 con	0.283	0.005	0.019	0.051	2.01	0.094	0.044	0.357	12	8.70	57.5
inc		−0.005									
100 con	0.301	0.008	0.015	0.049	2.43	0.043	0.042	0.333	12	11.4	60.3
inc		−0.008									
400 con-s	0.324	0.006	0.013	0.051	2.51	0.020	0.064	0.303	2	3.60	41.2
con-d	0.314	0.001	0.024	0.049	2.52	0.016	0.060	0.335	2	2.11	39.8
inc	0.320	-0.018	0.011	0.053	2.17	0.019	0.048	0.303	2	4.45	47.7

The values that differ for the incongruent condition, compared to the congruent (“con”) one, are given in the rows labeled as “inc”. For the 400-*ms* SOA condition “con-s” and “con-d” stand for “congruent-same” and “congruent-different”, respectively.

**Fig 8:**
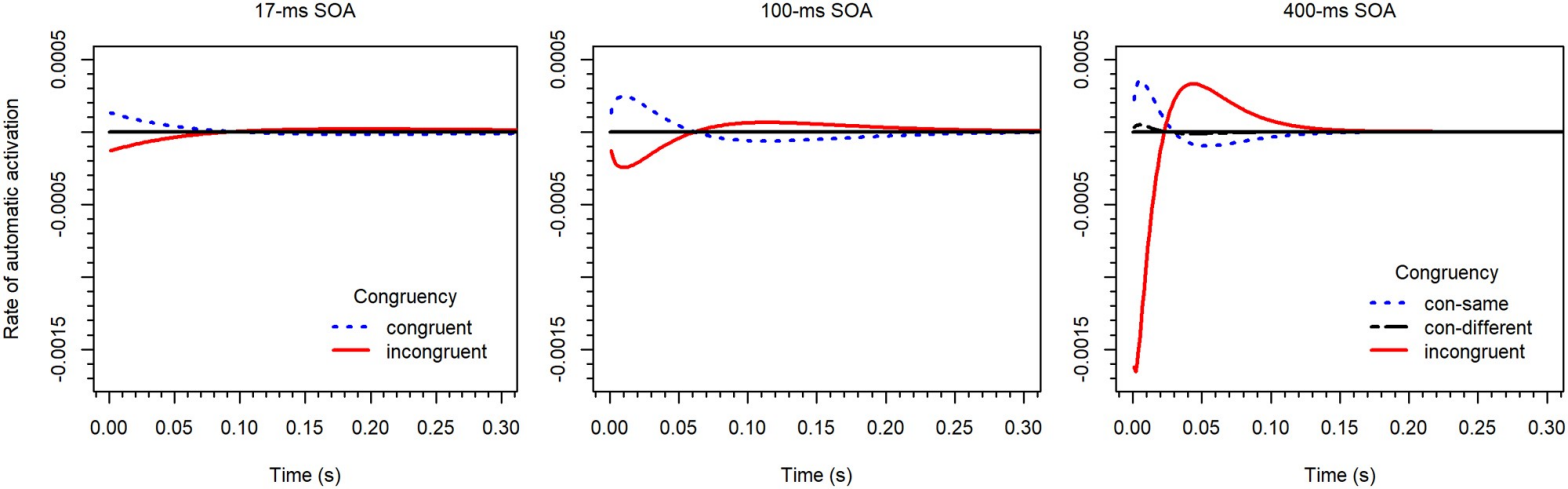
The rate (*μ*_*a*_(*t*)/1000) of the automatic process in the DMC model for the 17-*ms* (top left), the 100-*ms* (top right), and the 400-*ms* (bottom) SOA condition in Experiment 2.

The parameters of the Gamma distribution also varied considerably across the SOA conditions. The shape parameter *a* is somewhat smaller for the 17-*ms* SOA, compared to the other SOAs. In contrast, the parameter *τ* decreases with SOA. As consequence, the resulting rate functions for the automatic process differ substantially, as can be seen in Fig 8. As mentioned, by integrating the rate function *μ*_*a*_(*t*) for the automatic process one obtains the accumulated-rate function, which looks similar to an impulse (see Figure B1 in [15]). The maximum or peak of this impulse occurs at *t*_max_ = (*a*-1)*τ*.

As shown by Ulrich et al., the earlier the maximum is reached, the flatter the delta function. This is confirmed by our results (see also Fig 8). Since the shape parameter *a* did not vary much in size, the order of the *τ* values also reflects the order of the maxima. For incongruent stimuli in our 17, 100, and 400-*ms* SOA conditions the maxima occurred at 95, 61, and 22 *ms* after *t*_*0*_, respectively. Because the accumulated rate function represents the integrated rate, it follows that its maximum corresponds to the zero crossing of the rate function. This can also be seen in Fig 8, where the rate functions cross the zero line at the respective times before they reverse in sign.

A specific characteristic of the DMC model is that irrelevant activation is followed by inhibition, or vice versa. The reversal it important for reproducing the reversed congruency effects in accuracy for the two longer SOA conditions. Especially for incongruent stimuli in the 400-*ms* SOA condition, a large positive overshoot was necessary (Fig 8, lower panel). That is, after an initial activation of the wrong response the correct response was strongly activated over a relatively long period. However, the obligatory reversal in sign of activation after the zero crossing is not always appropriate. For instance, it is responsible for the mentioned underestimation of the congruency effect for slow responses in the 17-*ms* SOA condition (see Fig 6).

### The DSTP model

The main characteristics of the DSTP model [4] are two discrete stages of information selection, an early, and a late stage determining the rate of response selection. Response selection starts with the rate of evidence provided by Stage1. This stage is already selective, for instance by applying perceptual (e.g., spatial) filters, although selectivity is far from perfect. Therefore, a second and more effective stage of stimulus selection is assumed. If the processes at the first stage finish, the rate for response selection usually changes, which divides response selection into a first and a second phase (Phase 1 and Phase 2). Information selection at Stage 2 is also modelled by a diffusion process, running in parallel with response selection during Phase 1. If the evidence accumulated by this process in favor of some information C relative to information D hits threshold *C* or–*D*, then the rate of response selection changes to a corresponding value. However, it can also happen that a response is already selected during Phase 1.

To use the DSTP framework for modeling performance in a specific task, further assumptions have to be made. For modeling our data collected with the 17-*ms* SOA, we used our standard Flanker-task interpretation. For the other SOA conditions, we had to find a different interpretation.

#### Standard interpretation

For the standard Flanker task, it is assumed that the rate *μ*_*RS1*_ for the first phase of response selection is composed of two component rates, *μ*_*t*_, and *μ*_*f*_, which are the result of the early stage of stimulus selection. The components represent the evidence provided by the target and the flankers in favor of the correct response A, respectively. Both components sum up to the total rate, i.e. *μ*_*RS1*_ = *μ*_*t*_ + *μ*_*f*_. The component *μ*_*f*_ is positive, if the flankers are response compatible, but negative, if they are incompatible. Thus, the rate *μ*_*RS1*_ is usually smaller for incongruent than for congruent stimuli, and can even be negative.

To account for the fact that accuracy for incongruent stimuli usually improves with RT, the diffusion process that initiates a rate change of response selection is assumed to represent a late categorical stimulus-selection process *SS* with rate *μ*_*SS*_. It selects the mental category of the target letter or that of the flanker letter, depending on whether it hits thresholds *C* or–*D*, respectively. If the representation of the target letter is selected, then response selection continues with rate *μ*_*RS2C*_, which is usually higher, compared to *μ*_*RS1*_. In case the representation of the flanker letter was selected, the new rate is *μ*_*RS2D*_. This rate is positive or negative depending on whether the flanker is congruent or incongruent, respectively.

For the model applied to the 17-*ms* SOA condition in this study, we assumed symmetric thresholds for response and stimulus selection, i.e. *A* = *B*, and *C* = *D*. Furthermore, we assumed that target and flanker letter selection leads to the same rate for response selection in Phase 2, i.e., *μ*_*RS2C*_ = *μ*_*RS2D*_. Thus, altogether, the model has 7 parameters: Threshold *A* = *B* for response selection, the component rates for the target and flanker, *μ*_*t*_ and *μ*_*f*_, the rate *μ*_*RS2*_ for response selection in Phase 2, the rate, *μ*_*SS*_, and threshold *C* = *D* for the stimulus-selection process, and finally, a non-decisional time parameter, *t*_*nd*_.

**Fig 9:**
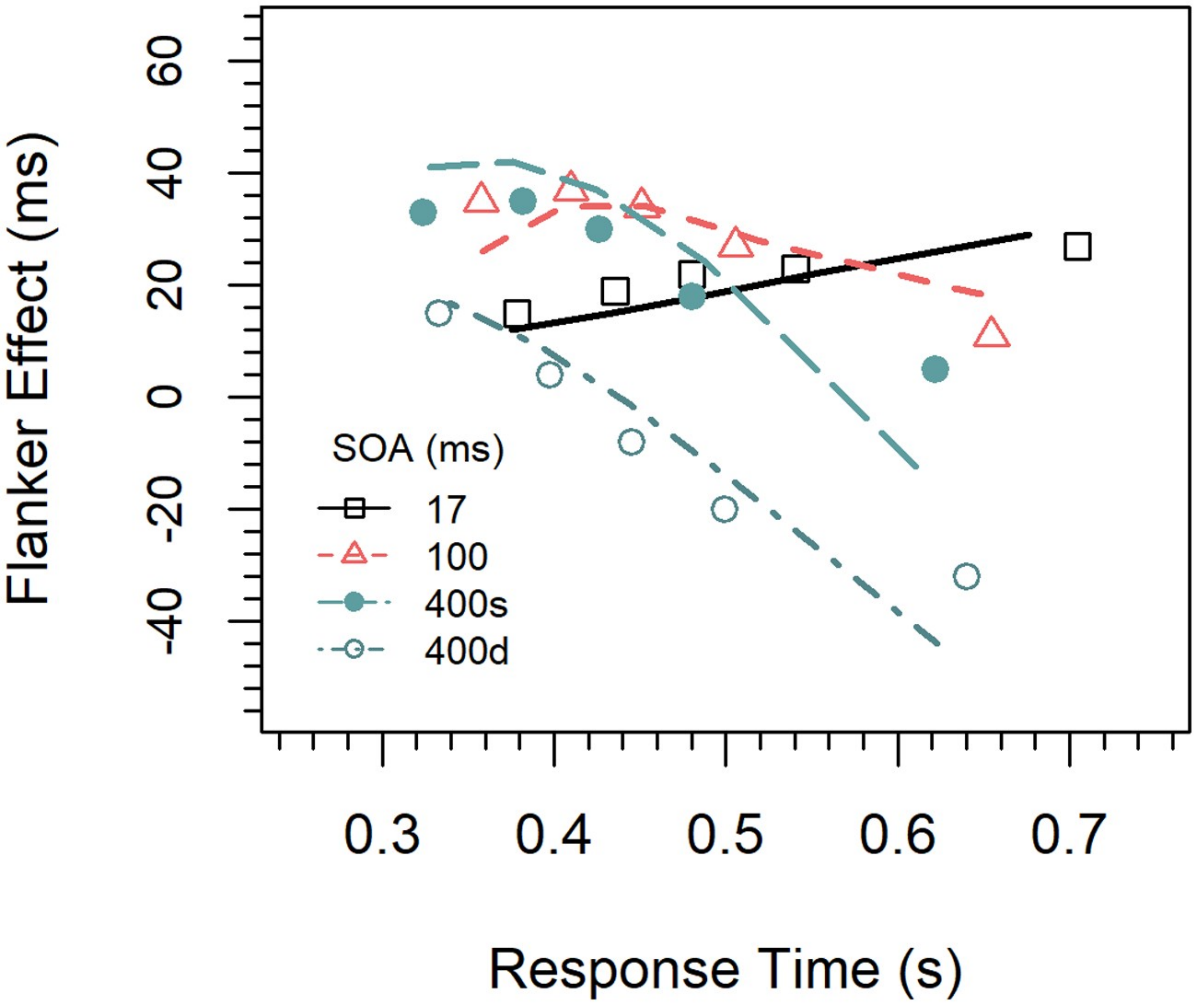
Delta functions for the different conditions in Experiment 2 (data points). The lines represent the fit of the DSTP model.

**Fig 10:**
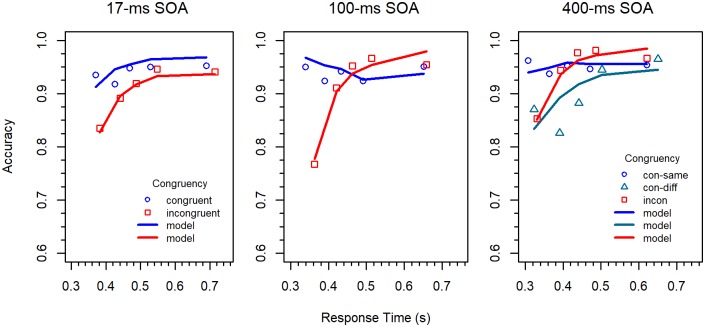
CAFs for the different conditions in Experiment 2 (data points). For the 17-ms and 100-ms SOA conditions, the data for congruent-same and congruent-different stimuli were collapsed. The lines represent the fit of the DSTP model.

This 7-parameter version of the DSTP model was fitted to our data from the 17-*ms* SOA condition with the same procedure as the DMC model. The resulting delta functions and CAFs are shown as line graphs in Figs 9 and 10, respectively, and the estimated parameter values are shown together with the goodness-of-fit measures in Table 2.

**Table 2:** Parameter estimates obtained by fitting the DSTP model to the distributional data of the different conditions in Experiment 2.

SOA (*ms*)	Parameters							*df*	*G*^*2*^	*BIC*
	*μ*_*RS1*_	*A*/*B*	*μ*_*SS*_	*C/D*	*μ*_*RS2C*_	*μ*_*RS2D*_	*t*_*nd*_			
17 con	0.117	0.072	0.303	0.079	1.017	1.017	0.248	13	13.4	56.1
inc	0.044					−1.017				
100 con	0.281	0.060	0.334	0.043	0.315	−0.532	0.273	12	11.8	60.7
inc	0.005				0.532	0.315				
400 con-s	0.228	0.057	0.322	0.050	0.313	0.016	0.236	3	2.39	35.3
con-d	0.122	0.057	0.331	0.053	0.363	−0.219	0.246	3	3.45	36.4
inc	0.091	0.060	0.343	0.044	0.485	0.426	0.258	3	6.89	44.7

The values that differ for the incongruent condition, compared to the congruent (“con”) one, are given in the rows labeled as “inc”. For the 400-*ms* SOA condition “con-s” and “con-d” stand for “congruent-same” and “congruent-different”, respectively.

As can be seen, the DSTP model nicely fits the delta function as well as the CAFs. Compared to the DMC model, the fit of the delta function is slightly better, whereas the fit to the CAFs is somewhat worse. If we consider *G*^*2*^, then the values are mostly larger for the DSTP model than for the DMC model. However, we also considered the Bayesian Information Criterion (BIC) model-selection statistics [43], which additionally takes the number of free model parameters into account. This statistics can easily be derived from the *G*^*2*^ statistics (cf. [42]) that we used for data fitting. The best model is the one with the smallest BIC. Obviously, in the present case the BIC is mostly smaller for the DSTP model.

If we consider the rates for response selection in Phase1, then we see that *μ*_*RS1*_ is larger for congruent than for incongruent stimuli, which indicates the effect of early selection (perceptual filtering). After late target selection (Phase 2), the rate *μ*_*RS2C*_ is substantially increased, which is typical for this task [4].

Because the 17-*ms* condition is quite similar to the standard flanker task, it is no surprise that the DSTP model fits the corresponding data well. However, with this interpretation the model accounts neither for the negatively sloped delta functions nor for the reversed flanker effects. Thus, for the two longer SOA conditions we had to adapt the model accordingly.

#### Different interpretation

As our data show, if flankers appear far ahead of the target, they have strongly activated their associated response before evidence accumulation can start. Thus, to prevent premature responding and limit the number of fast errors, this activation is presumably suppressed. However, if suppression takes some time to come into effect, as is usually assumed [10], then, with respect to our model, its major effect should occur in Phase 2 and be reflected by the corresponding rates. For relatively long SOAs, however, activation suppression might also already affect the rate for response selection in Phase 1.

For the 100-*ms* SOA condition, our data indicate that the response activated by the flankers was generally suppressed. In case of incongruent flankers, this means that the incorrect response was suppressed, which was appropriate. However, if the flankers were congruent, then the correct response was suppressed (remember that we do not differentiate between congruent-same and congruent-different for this SOA condition). Accordingly, we assumed that, given the target category is selected, suppression leads to a rate *μ*_*RS2C*_ that is smaller for congruent than for incongruent stimuli. Thus, different from the standard flanker-task, the rate *μ*_*RS2C*_ in Phase 2 is no longer identical for the different congruency types. Consequently, we had to introduce an additional parameter to allow individual rates *μ*_*RS2C*_ for congruent and incongruent stimuli, respectively.

Whereas a lower rate for congruent stimuli in Phase 2 already produces negatively sloped delta functions, it was not sufficient for also producing the relatively low accuracy for congruent stimuli in the midrange bins of the corresponding CAFs, i.e., the reversed congruency effect. The shapes of the CAFs seem to reflect specific errors, which are not taken into account by the delta functions, because errors are excluded for computing the cumulative RT distribution functions.

Which mechanism could have produced the relatively low accuracy for the responses to congruent stimuli? A possible idea is to assume that the reversed flanker effect was due to some specific inhibition on trials on which the participants mistakenly responded to the flankers. Several corresponding mechanisms, such as *negative priming* [44, 45], especially *spatial* negative priming [46] have been proposed. It is usually assumed that representations competing with the action goal are selectively inhibited [47]. That responding to stimuli at an inhibited location can also be impaired in a flanker task has already been shown [48, 49]. Furthermore, it is even conceivable that an erroneous selection of flanker category triggers an ‘emergency break’ [50, 51], leading to a strong inhibition of the response associated with the flankers.

In any case, if one assumes that, in addition to suppressing irrelevant response activation, the processing of items at the flanker positions is additionally inhibited, then selecting a congruent flanker category leads to a negative rate *μ*_*RS2D*_ for response selection. In contrast, if an incongruent flanker category is selected, its inhibition leads to a positive *μ*_*RS2D*_. Together, this produces a reversed congruency effect, especially in the CAFs.

This specific interpretation of the DSTP model was first fitted to the data from the 100-*ms* SOA condition. To limit the number of free parameters, we assumed that for incongruent stimuli, *μ*_*RS2D*_ is the same as −*μ*_*RS2C*_ for congruent stimuli, and that *μ*_*RS2D*_ for incongruent stimuli is identical to *μ*_*RS2C*_ for congruent ones. With these assumptions, the DSTP model has eight parameters. This version was fitted simultaneously to our data from the congruent and incongruent conditions, where congruent-same and congruent-different data were collapsed.

As can be seen in Figs 9 and 10, the fit to the delta functions and CAFs is rather good. The parameters in Table 2 show that the rates for early selection (*μ*_*RS1*_) differ more between congruent and incongruent stimuli than the corresponding rates for the 17-*ms* SOA condition. This reflects the fact that, despite activation suppression, the flankers had a larger effect in the 100-*ms* SOA condition, which is particularly pronounced in the error rates for fast responses. Moreover, the rates for late selection are considerably reduced, compared to the 17-*ms* SOA condition, indicating general suppression. However, as expected, *μ*_*RS2C*_ was smaller for congruent than for incongruent stimuli. Taken together, the 8-parameter DSTP model accounts well for the 100-*ms* SOA condition.

In the 400-*ms* SOA condition, suppression had a relatively long period to be effective before the target appeared. Moreover, after target onset, participants were able to detect whether the stimulus was congruent-same or not, and to adapt activation suppression accordingly. Because these mechanisms produced large differences in performance for the two congruent stimulus types, we modelled these conditions separately, as for the DMC model.

The fit results are shown in Figs 9 and 10, and the corresponding parameters are listed in Table 2. As can be seen, already the rate *μ*_*RS1*_ for response selection in Phase 1 differs largely between congruent-same, congruent-different, and incongruent. Obviously, due to the long SOA, suppression had time to diminish the influence of the flankers in Phase 1, which reduced the negative effect of incongruent flankers, but also the benefit from congruent flankers. Compared to the congruent condition for the 100-ms SOA, the benefit, i.e. *μ*_*RS1*_, was reduced for congruent-different stimuli, whereas the rate for congruent-same stimuli was hardly affected.

If we consider the rate *μ*_*RS2C*_ in Phase 2, then we see that it is larger for incongruent than for the two congruent stimulus types, which is comparable to the 100-*ms* SOA condition. With respect to *μ*_*RS2D*_, we see that it is again negative, but only for congruent-different. For congruent same, this rate was small but positive, indicating that processing the information at the flanker position was less inhibited if it was detected that the stimulus was congruent.

### Discussion

Our modeling revealed that both the DMC and the DSTP model account well for our data from Experiment 2. It also became clear that passive decay of response activation alone is not sufficient for explaining the data, especially not the reversed congruency effects in RT and accuracy for the longest SOA. To model the performance for incongruent stimuli in the 400-*ms* SOA condition, both models need some kind of rate reversal during the course of response selection. A reasonable idea is to assume some kind of suppression or inhibition that not only reduces the effect of irrelevant activation, but also, after some time of impact, produces the opposite effect. In the DMC model, this is achieved by an intensive positive overshoot after the initial period of activating the wrong response.

For the DSTP model, we assumed that response suppression was effective in Phase 2 of response selection, i.e., after late stimulus selection. Its effect is reflected by a smaller rate for congruent than for incongruent stimuli. Moreover, to account for the reversed congruency effect in the CAFs, we assumed that on some trials, probably on those where flanker letter category had mistakenly been selected by late selection, stimulus processing was strongly inhibited.

Taken together, our modeling shows that current drift-diffusion models can successfully account for negatively sloped delta functions and reversed congruency effects. Even though the architecture of the DMC and the DSTP model are rather different, both fit the data similarly well.

## Experiment 3 (Simon task)

So far, we have shown that the flanker task can be used to produce similar data as the Simon task. For specific delays between irrelevant and relevant information there were not only negatively sloped delta functions but also negative flanker effects. However, are our data indeed similar to those obtained with the Simon task? To see whether this is the case, we conducted a Simon-task experiment. Also for this task, we wanted to have some modulation of the congruency effect. Because the standard Simon task already includes a temporal distance between irrelevant and relevant information, we thought that further varying this distance would make little sense for our objective. Moreover, in a former paper from our group [7], we have already shown that a further modulation of the temporal distance affects the offset of the delta function more than the slope.

Therefore, we decided to vary the proportion of congruent trials relative to that of incongruent ones. Although this variation has been realized in various Simon-task experiments before (e.g., [20, 52–55]), in none of these the delta functions were analyzed. If *proportion congruent* (PC) is high (HPC), then the Simon effect is usually increased compared to when both trial types are balanced. In contrast, if PC is low (LPC), then the effect is smaller (e.g., [55]) and can even become negative (e.g., [54]). In the present experiment, PC was 75% or 25%, respectively. We expected that the delta functions would be negatively sloped, but that the slope varies with PC. In any case, the DMC and DSTP models as applied in Experiment 2 should again account well for the data.

### Method

Participants. Students from the Universität Konstanz participated in the experiment. All had normal or corrected-to-normal vision, and were paid 5 € for their participation in a ½-hr session. The HPC and LPC conditions were administered to a group of 11 participants (mean age of 23 years; 1 male), and a group of 10 students (mean age of 21 years; 2 male), respectively.

Apparatus and stimuli. The apparatus was the same as in the other experiments. Stimuli were red and blue squares of 2.2×2.2 cm presented against a black background. The squares appeared randomly at 2.1° left or right of fixation.

Procedure. Each trial started with the presentation of a fixation cross at the center of the screen for 400 *ms*, followed by a blank screen for 400 *ms*. Then the target square appeared for 165 *ms*. The screen remained blank until response. After the response, a blank screen was shown for 1000 *ms* before the next trial started. The task was to indicate the color of the square by pressing one of the two mouse buttons (left button for blue; right button for red) with the index or middle finger of the right hand, respectively. The participants were instructed to respond as fast as possible without making many errors. Errors were signaled by a beep. In addition to a practice block, there were 10 experimental blocks of 64 trials. HPC and LPC blocks contained 75% and 25% congruent trials, respectively.

### Results

Responses faster than 100 *ms* or slower than 1200 *ms* were excluded from analysis (< 0.8% of all data).

#### Response times

Mean response time of correct responses was 413 *ms* (*SD* = 62.0). The RTs of correct responses were subjected to an ANOVA with the within-participant factor *congruency* (congruent, or incongruent), and the between-participants factor *proportion* (HPC, or LPC). The analysis revealed a main effect of *congruency*, *F*(1, 19) = 149, *p* < .001, η_p_^2^ = .887. Responses to congruent stimuli were faster than those to incongruent ones [390 *ms* (*SD* = 59.8) vs. 437 *ms* (*SD* = 55.9)]. However, there was also a significant interaction between *congruency* and *proportion*, *F*(1, 19) = 63.5, *p* < .001, η_p_^2^ = .770. It indicates that the Simon effect was stronger in the HPC condition [369 *ms* (*SD* = 26.8) vs. 446 *ms* (*SD* = 32.8)] than in the LPC condition [412 *ms* (*SD* = 78.1) vs. 427 *ms* (*SD* = 74.2)].

#### Error rates

Mean error rate was 8.49% (*SD* = 7.49). Subjecting the error rates to an ANOVA of the same type as for the latencies revealed significant main effects of *congruency*, *F*(1, 19) = 65.8, *p* < .001, η_p_^2^ = .776, and *proportion*, *F*(1, 19) = 12.3, *p* < .01, η_p_^2^ = .393. Responses were more reliable on congruent trials than on incongruent trials (3.80% vs. 13.2%), and in the LPC condition, compared to the HPC condition [6.30% (*SD* = 9.57) vs. 10.5% (*SD* = 3.18)]. However, there was also a significant interaction between *proportion* and *congruency*, *F*(1, 19) = 45.8, *p* < .001, η_p_^2^ = .710, indicating a larger Simon effect in the HPC condition [2.07% (*SD* = 1.30)vs. 18.9% (*SD* = 5.89)] than in the LPC condition [5.71% (*SD* = 4.02) vs. 6.88% (*SD* = 2.11)].

#### Distributional analyses

**Fig 11:**
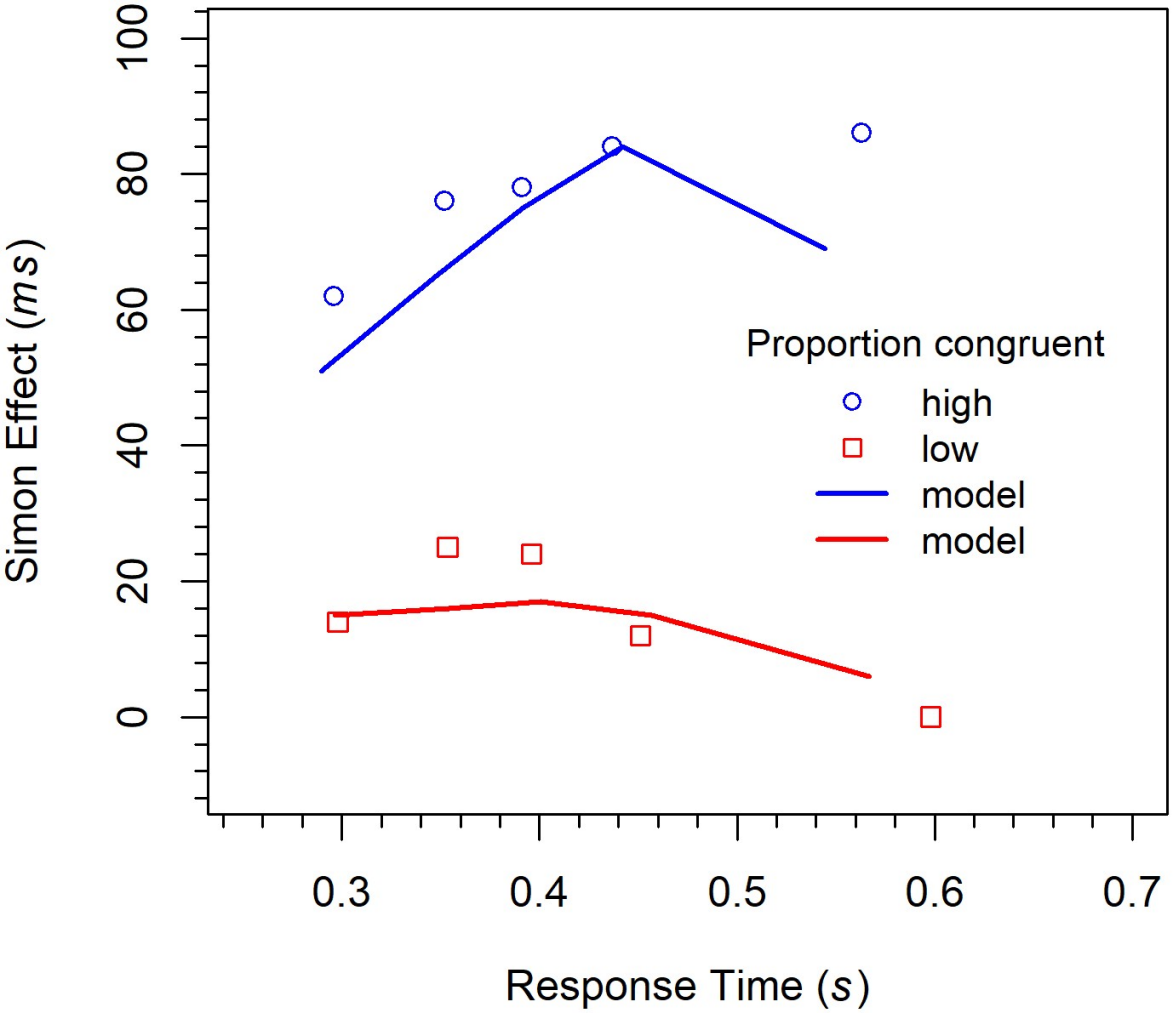
Delta functions for the different conditions in Experiment 3 (Simon task). The lines represent the fit of the DMC model.

Delta functions. Delta functions were computed and analyzed analogously to those in the previous experiments. The functions are shown in Fig 11 together with one model fits. Their analysis revealed a significant three-way interaction between *quintile*, *congruency*, and *PC*, *F*(4, 76) = 2.86, *p* < 0.05, η_p_^2^ = .131. As can be seen in the corresponding figures, the slope of the delta function for the HPC condition is positive, whereas that for the LPC condition is first positive, but then turns negative. To analyze the data further, we also computed the slopes individually for each participant and condition. For the HPC condition, simple linear regression revealed that the slope (0.445) was significantly greater than zero, *t*(10) = 2.28, *p* < 0.05, Cohen’s *d* = 0.689. Because simple linear regression was obviously inappropriate for the LPC condition, we fitted a polynomial of degree 2 to the data. It revealed that the coefficient of the linear term (0.163) did not differ significantly from zero, *t*(9) = 0.570, *p* = 0.583, *d* = 0.180. However, the coefficient of the quadratic term was significantly smaller than zero *t*(9) = -1.97, *p* < 0.05 (one-sided), *d* = -0.624, indicating that the delta function was positive-going for the faster responses but negative-going for the slower ones. Concerning the linear term, power analysis showed that in order for an effect of this size to be detected (80% chance) as significant at the 5% level (one-sided), a samples of 192 participants would be required.

**Fig 12:**
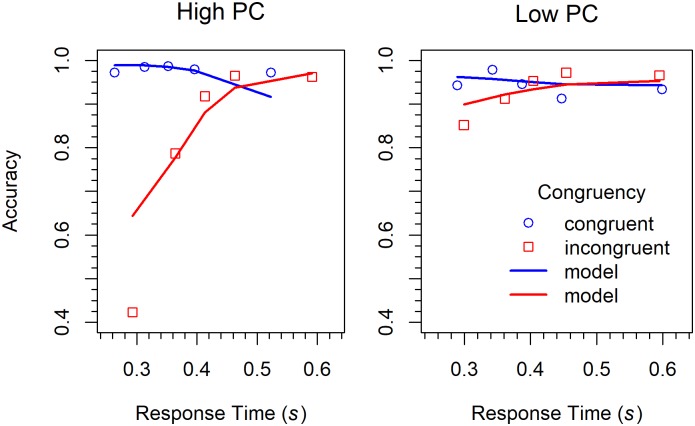
Conditional accuracy functions for the different conditions in Experiment 3 (Simon task). The lines represent the fit of the DMC model.

Conditional accuracy functions. CAFs were computed in the same was as in Experiment 1. The results are shown in Fig 12 together with a model fit. As can be seen, in the LPC condition there was a reversal of the Simon effect in the medium range of RTs, which was absent in the HPC condition.

### Modeling

The similarity between the SOA flanker task and the Simon task should also be demonstrated by modeling. Because the present results are rather similar to those obtained for the longer SOAs in Experiment 2, the corresponding model types were applied.

#### DMC model

The DMC model was fitted with the same procedure and in the same way as in Experiment 2. The delta functions are shown in Fig 11, where model performance is represented by the line graphs. As can be seen, the fit is not as good as for the SOA flanker task. However, deviations from the data are mainly present for the slower responses. Concerning the CAFs (see Fig 12), there is mainly a large deviation for fast responses to incongruent stimuli in the HPC condition.

The corresponding parameter values are listed in Table 3. As one would have expected, the contribution of automatic processes was much smaller in the LPC than in the HPC condition (see parameter *m*). The shape parameter *a* also differs largely between the conditions. Furthermore, there is a relatively large difference in *σ*_*start*_. For a drift-diffusion process, an increased variability of the starting value reduces the accuracy of fast responses [38]. This was also the case in the HPC condition, i.e., the error rate was increased. However, this was not sufficient to reproduce the extremely low accuracy for the fastest responses.

**Table 3:** Estimated parameter values obtained by fitting the DMC model to the data of Experiment 3.

PC	Parameters								*df*	*G*^*2*^	*BIC*
	*μ*_*c*_	*m*	*σ*_*start*_	*A*/*B*	*a*	*τ*	*σ*_*tnd*_	*t*_*nd*_			
HPC con	0.308	0.021	0.123	0.047	2.688	0.058	0.054	0.274	12	14.2	65.0
inc		−0.021									
LPC con	0.305	0.006	0.007	0.046	1.518	0.082	0.054	0.283	12	16.1	66.9
inc		−0.006									

The values that differ for the incongruent condition, compared to the congruent (“con”) one, are given in the rows labeled as “inc”.

**Fig 13:**
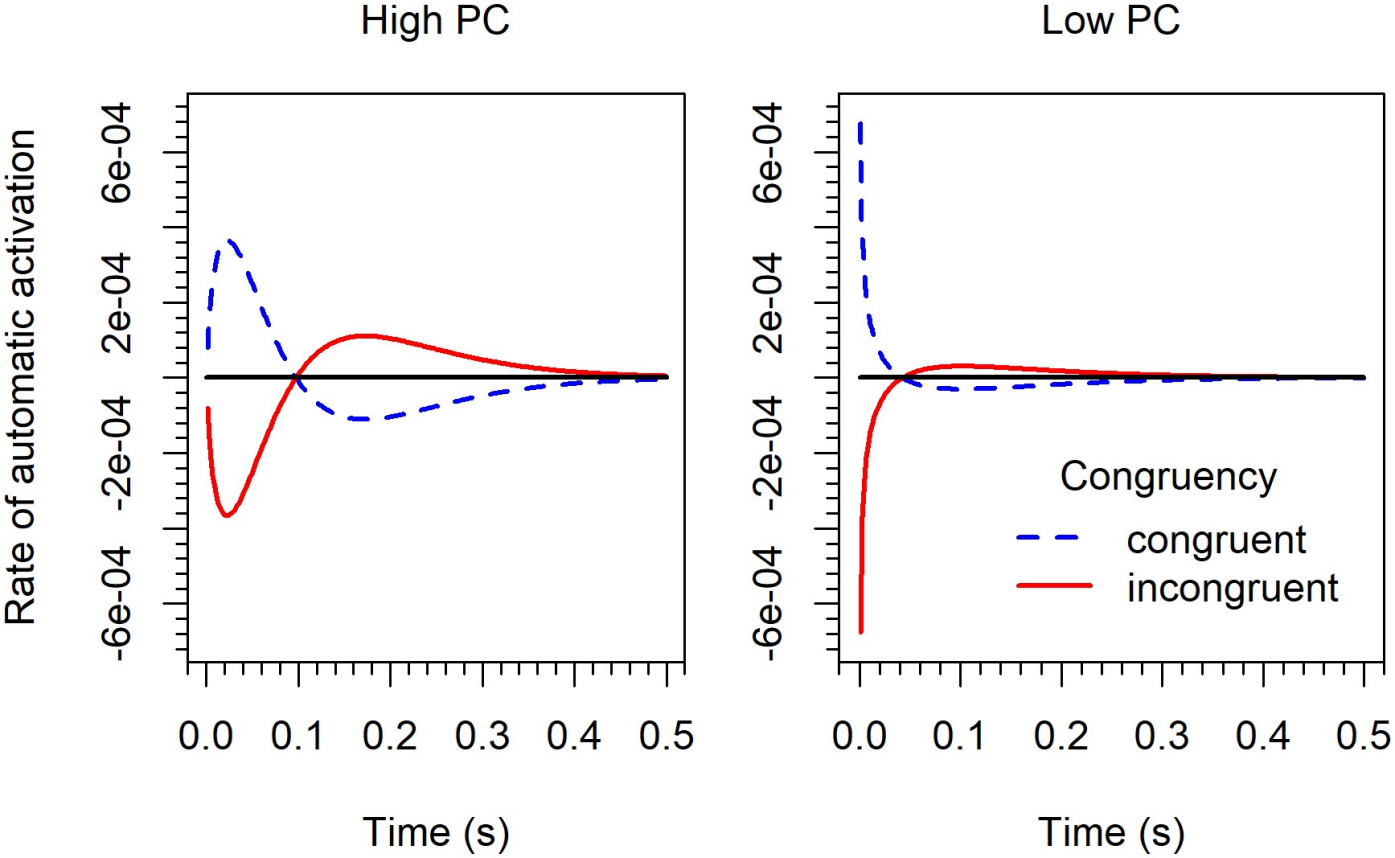
The rate (*μ*_*a*_(*t*)/1000) of the automatic process in the DMC model for the LPC and the HPC condition in Experiment 3 (Simon task).

The accumulated-rate functions for the LPC and HPC condition have their maximum at 43 and 98 *ms* after *t*_*0*_, respectively. Thus, also for these data the peak times systematically relate to the slope of the delta functions. If we consider the corresponding rate functions (see Fig 13), then we see that the irrelevant activation for the LPC condition is relatively high immediately after stimulus onset, but then decays quickly and crosses the null line. In contrast, activation for the HPC condition builds up and decays more slowly. The relatively slow activation is necessary for obtaining a positively sloped delta function. However, this phase is followed by a large overshoot, which produces a reduction and reversal of the congruency effect for slow responses in the latencies and accuracy, respectively, which were not present in the data.

#### DSTP model

For modeling the SOA flanker task with the DSTP model, we interpreted the possible rate parameters in Phase 2 of response selection in a specific way. Especially the negative rate *μ*_*RS2D*_ was interpreted as reflecting inhibition of processing at the flanker location, if a flanker had erroneously been selected. How can we interpret this parameter for the Simon task, where we have no flankers? We simply assumed that on some trials the late selection process erroneously select *stimulus location* instead of the relevant stimulus feature. As consequence, a specific location-based inhibition (‘emergency break’ [50, 51]) was initiated, or some extra suppression of the response associated with stimulus location. Inhibition is assumed to be stronger the greater the probability that this mistake will produce an error.

**Fig 14:**
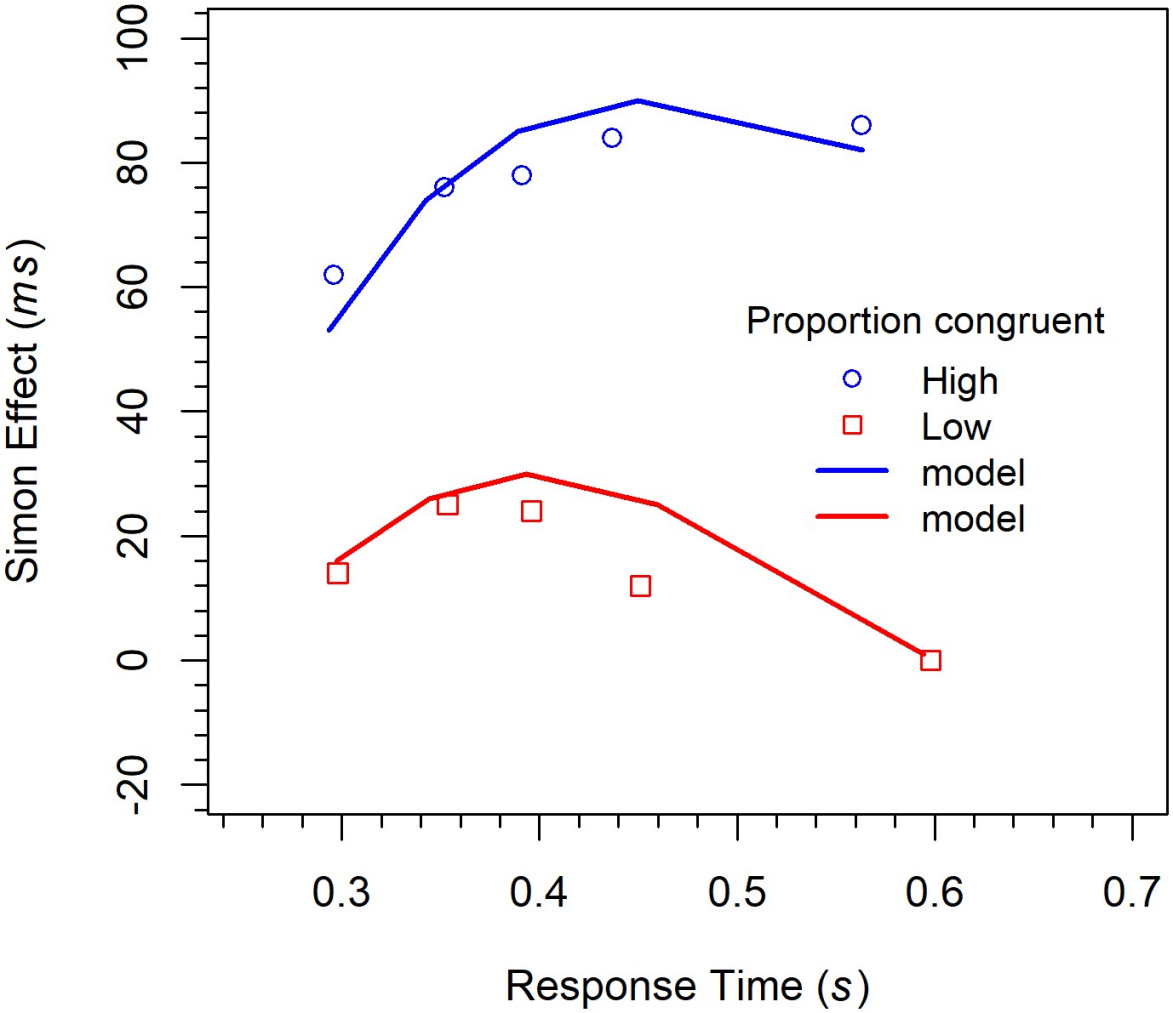
Delta functions for the different conditions in Experiment 3 (Simon task) and the fit (lines) of the DSTP model.

**Fig 15:**
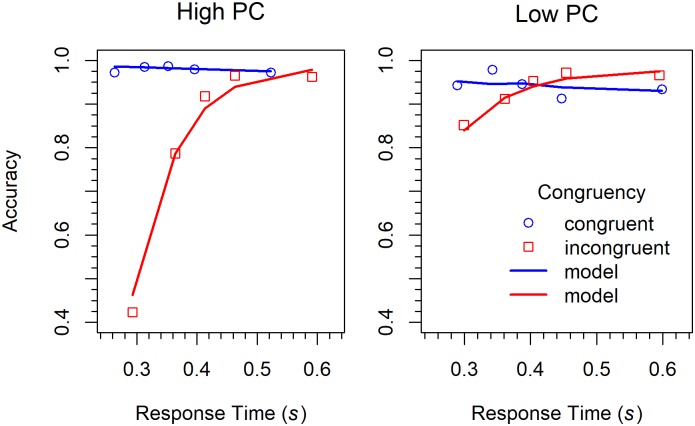
Conditional accuracy functions for the different conditions in Experiment 3 (Simon task) and the fit (lines) of the DSTP model.

Irrespective of whether this interpretation is valid or not, we could apply the DSTP model in the same way as in Experiment 2. The fits to the delta functions and to the CAFs are shown in Figs 14 and 15, respectively. Obviously, the model also accounts for the data rather well. If we consider the parameters, then we see that for incongruent stimuli in the HPC condition the rate in Phase 1 is even negative, indicating a rather large and early influence of stimulus location on response selection in this condition.

The rates in Phase 2 show a similar pattern as those for the SOA flanker task. The rates for congruent stimuli are smaller than the rates for incongruent ones, reflecting response suppression. For the LPC condition, the rates are generally smaller compared to the HPC condition, indicating an overall higher level of response suppression and inhibition if incongruent trials are frequent.

### Discussion

The results of this experiment show that the delta functions obtained with the Simon task are not generally negatively sloped. Rather, the slope strongly depends on the specific conditions (see also [56]). Here, it was positive when the proportion of congruent stimuli was high. When the proportion was low, i.e. when a high level of control was required, then the slope was negative even if only for the slower responses. This demonstrates that the slope of the delta functions cannot only be manipulated by varying the temporal distance, but also by changing the control demands, in our case by modulating the utility of the irrelevant information in a block of trials. Thus, even if irrelevant spatial information might generally be processed faster than relevant stimulus features, there is still much room for control mechanisms to modulate the effects of this temporal advantage, for instance, by inhibiting the processing of irrelevant information and by suppressing response activation that still leaks through. If the control effort exceeds a certain strength, then a negatively sloped delta function results, or even a reversed congruency effect. This is also the case if one assumes that at least part of the variation of the congruency effect with PC is due to sequential effects [12, 53, 56, 57].

That our Simon-task data are similar to the data obtained in our SOA flanker-task experiments is confirmed by our modeling results. In the DMC model, the differently sloped delta functions for the HPC and LPC conditions, respectively, were again produced by corresponding late and early zero crossings of the rate function for the automatic activation. Although the overall fit was rather good, the DMC model has difficulties to fit simultaneously specific details of the delta function and CAFs. If we consider the fit in Fig 11, then we see that the estimated initial rate for the HPC condition is not large enough to reduce accuracy below 50% for fast responses. On the other hand, the resulting large overshoot produced a small reversal of the congruency effect in accuracy, which is absent in the data. The overshoot also caused the underestimation of the congruency effect for the last point in the delta function for the HPC condition. These difficulties result from the characteristic of the DMC model that irrelevant activation is followed by inhibition, or vice versa. More specifically, for producing a delta function with a positive slope the null line crossing must occur relatively late, while the irrelevant activation has to remain sufficiently high. Shifting the null-line crossing to later times by increasing parameter *τ* also largely reduces the maximum rate of activation. Therefore, as we can see in Table 3, the shape parameter *a* is increased instead. This, however, leads to a large overshoot (Fig 12), which reduces the congruency effect for slow responses (Fig 13).

For the LPC condition, the situation is the opposite. Here, irrelevant activation is effective only for a short period directly after stimulus onset. In this case, however, the overshoot could have been larger to reproduce the reversed congruency effect in accuracy for the slower responses (see the right panel in Fig 11).

These analyses show that the data are the result of a complex interaction between activations and adaptive control processes. Simply assuming that automatic activation occurs earlier for the Simon task than for the flanker task, because stimulus location is processed faster than other stimulus features, is not sufficient.

The DSTP model also fitted the data well. Because the relation between irrelevant activation and its suppression is more flexible in this model, the fits seem visually to be somewhat better than those for the DMC model. However, the goodness-of-fit measures (Tables 3 and 4) indicate that the DMC model is superior, especially for the HPC condition.

**Table 4:** Parameter estimates obtained by fitting the DSTP model to the distributional data of the different conditions in Experiment 2.

PC	Parameters							*df*	*G*^*2*^	*BIC*
	*μ*_*RS1*_	*A*/*B*	*μ*_*SS*_	*C/D*	*μ*_*RS2C*_	*μ*_*RS2D*_	*t*_*nd*_			
HPC con	0.333	0.061	0.430	0.053	0.360	−0.450	0.199	12	21.2	72.0
incon	−0.133				0.450	0.360				
LPC con	0.270	0.055	0.414	0.046	0.271	−0.362	0.226	12	17.6	68.3
Incon	0.106				0.362	0.271				

The values that differ for the incongruent condition, compared to the congruent (“con”) one, are given in the rows labeled as “inc”.

## General discussion

The aim of the present study was to investigate whether the flanker task can be modified in such a way that the performance is similar to that usually observed in the Simon task. In various experiments, it has been observed that the congruency effect in the latencies increases with RT for the flanker task, but decreases for the Simon task, which is often expressed by positively and negatively sloped delta functions (e.g., [11]), respectively. A widely acknowledged explanation for this phenomenon is to assume that the temporal distance between irrelevant and relevant response activation is larger for the Simon task than for the flanker task (e.g., [9, 13]).

If the temporal distance between the activations is indeed the crucial difference between the two experimental paradigms, then it should also be possible to produce negatively sloped delta functions with the flanker task by presenting the flankers ahead of the target. Although such SOA experiments have been conducted before [27, 28, 58], only one considered RT distributions [27], which, however, were not analyzed appropriately for our objective. Therefore, we conducted two own SOA experiments. For direct comparison, we also conducted a Simon-task experiment. The data of our second flanker-task experiment were as expected. Therefore, they and the data of our Simon-task experiment were also modeled with drift-diffusion models to see whether the same model can account for the results in both paradigms.

### Experiments

In our first flanker experiment, participants had to categorize which of two letters appeared at target position. To test how the delta functions vary with the temporal distance between irrelevant and relevant information, we varied the (negative) SOA (17, 100, and 400 *ms*) between target and flankers. The results of the mean RT and the error rates of our experiments were similar to previous findings [27, 28, 58]. The congruency effect was largest for the 100-*ms* SOA and smallest for the 17-*ms* SOA, which indicates that the flankers had different effects, depending on the SOA.

Different from our expectation, though, the delta functions were more flat than decreasing, even for the 400-*ms* SOAs. After reconsidering our method, we hypothesized that the reason could have been that congruent and incongruent stimuli always consisted of identical and different letters, respectively. Our participants presumably used this difference to detect whether a stimulus was congruent or not, and adapted their control effort accordingly. That is, they suppressed irrelevant activation only on incongruent trials, which is optimal and leads to relatively flat delta functions.

This idea was tested in our second experiment, where two letters were mapped to each response button. Consequently, some congruent stimuli consisted of identical letters (congruent same), whereas others included different ones (congruent different). As result, we found similar effects for congruent-same stimuli as in Experiment 1. Congruent-different stimuli, however, produced different effects. For the longest SOA there was even a negative congruency effect in the latencies as well as in the error rates.

These data clearly indicate that there was some kind of suppression. Furthermore, the fact that the results differ between congruent-same and congruent-different demonstrates that participants are not only able to quickly detect equality of letters, but also to adapt their activation suppression accordingly on-the-fly. For the Simon task such flexibility has been shown before [7]. Importantly, though, our results show that the slope of delta functions can be modulated within the flanker task by varying the temporal distance between irrelevant and relevant response activation. For the shortest SOA we observed increasing delta functions, which is typical for the standard flanker task (e.g., [4, 12, 14]). In contrast, for the longer SOAs the delta functions in the congruent-different condition were negatively sloped, as is usually observed for Simon-task (e.g., [14, 18, 19, 59]). These results demonstrate that the crucial difference between the flanker task and the Simon task is the different time overlap between irrelevant and relevant reaction activations. However, for producing negatively sloped delta functions it is also important that the control demands require a strong suppression of irrelevant response activation.

For providing direct evidence for the similarity between the Simon task and the SOA flanker task, we further conducted a Simon-task experiment. To have also some modulation of the congruency effect, we varied the proportion of congruent (PC) stimuli. As result, the slope of the delta function was negative-going, at least for slow responses, only when PC was low. This demonstrates that even for the horizontal Simon task the delta functions are not always negatively sloped. Rather, the slope depends on the specific conditions [56].

Thus, our experiments demonstrate that typical Simon-task data (in particular, negatively sloped delta functions) can also be produced with the flanker task and that flanker-task like data (in particular, positively sloped delta functions) can be obtained with the Simon task as well. Taken together, our results suggest that the temporal distance between the irrelevant and relevant response activation is necessary, but obviously not sufficient for producing negatively sloped delta functions. What is equally important is the presence of control demands that require the strong suppression of irrelevant response activation. Thus, the slope of delta functions can be manipulated by varying the temporal distance between the activations and/or by varying the control demands.

### Modeling

To get an idea of what control mechanisms might have been involved in our experiments, we fitted the DMC model [15] and the DSTP model [4] to the data of Experiments 2 and 3. The DMC fitted the flanker-task data well. The resulting parameters reveal that already in the 100-*ms* SOA condition, there was strong suppression. That is, after its initial increase the rate of irrelevant activation crosses the zero line and reverses in sign for some period before it finally approaches zero. For incongruent stimuli in the 400-*ms* SOA condition, the amplitude of this damped oscillation was even dramatically large (Fig 8). A large overshoot is necessary for producing reversed congruency effects in RT and accuracy (see also [15]).

The DMC model also fitted the data collected with Simon task data well, except that for the HPC condition accuracy for fast responses to incongruent stimuli was overestimated, and the corresponding congruency effect in RT for slow response was underestimated. These deviations reflect the specific characteristic of the model that the delta functions are positively or negatively sloped, depending on when the irrelevant rate reverses in sign. The later this happens the steeper the delta function. However, the time of reversal also affects the size of the overshoot. As can be seen in Figs 11 and 12, the relatively late reversal for the HPC condition goes along with a subsequent large overshoot, which reduces the congruency effect in RT for slow responses and even produces some reversed congruency effect in accuracy, which deviates from the data.

In contrast to the DMC model, the DSTP model assumes that the rate changes abruptly during response selection due to a late stimulus selection process. Depending on the selected stimulus feature, the rate can take on different values. With these original assumptions, the model can well account for flanker-task data [4, 32]. Accordingly, for the 17-*ms* SOA condition, which produced typical flanker-task data, we could simply apply the standard interpretation of the model. Although it has been speculated that the DSTP model cannot account for negatively sloped delta functions [34, 36], here, we have demonstrated that this is not the case. By interpreting the late-selection mechanism in a slightly different way, the DSTP model can also produce negatively sloped delta functions.

We assumed that the late-selection process does not represent a specific type of stimulus selection, but rather a general process that alters the rate, where the new rate can result from various sources. For instance, to implement activation suppression, we assumed that the late rate for response selection is lower for congruent than for incongruent stimuli. This makes sense, because, if flankers are generally suppressed, then, in case of a congruent stimulus, the correct response is suppressed. This assumption was sufficient for obtaining negatively sloped delta functions. However, it could not produce reversed congruency effects in accuracy, as observed for the longer SOAs.

Although the reversed congruency effects are due to only a relatively small proportion of trials, they nevertheless seem to indicate a complex control mechanism. Here, we assumed that, in addition to a general suppression of response activation, responding to information at flanker location is also inhibited. Such inhibition has been proposed in other areas as well. Examples are spatial negative priming [46], or masked priming [60]. In our case, we assumed that such an inhibition has an effect only in the rare cases when the flanker category is erroneously selected by late selection. If the stimulus is congruent, this inhibition most likely produces an error. In contrast, for incongruent stimuli, it produces a correct response. Together, these effects result in a reversed congruency effect.

The version of the DSTP model that had successfully been applied to the data from the longer SOA flanker conditions was also fitted to the Simon-task data. Because there is no flanker selection in this task, we assumed that stimulus location corresponds to flanker location. That is, the task of late stimulus selection now is to select relevant stimulus features against the impact of stimulus location. However, because selection is not perfect, on some trials location is mistakenly selected. Like in the SOA flanker task, the processing of location information is then inhibited. It is also conceivable that in this case participants are reluctant to respond to the selected location (emergency break [50, 51]). In any case, this inhibition adds to activation suppression and leads to reversed congruency effects. Although these scenarios are speculative, they are not unlikely. In any case, with this interpretation the DSTP model fitted the data well.

Taken together, our modeling with drift-diffusion models was rather successful. Despite their different architecture, both the DMC as well as the DSTP models account well for the data. This is in accord with the theoretical analyses of Schwarz, Miller [61], who found that diverse models with different processing architectures can be consistent with negatively sloped delta functions.

The rate function of the DMC model turned out to be rather flexible in adapting to different conditions. It seems that merely the relation between the time and amplitude of activation reversal poses some limit. Moreover, the DMC model is not very specific about the mechanisms producing the variation of the rate. The DSTP model is more specific, although the proposed mechanisms for the present case are rather speculative.

The high complexity of the DSTP model makes data fitting relatively difficult. Recently, it has been stated that parameter recovery is worse for the DSTP model than for the DMC model [36]. We doubt that this holds generally. Because the DSTP model is a mixture model, some events can occur very infrequently. Therefore, we usually simulate 8·10^5^ trials for each fit iteration. In contrast, White et al. only used 10^4^ trials.

### Conclusions

Our experiments as well as our modeling provide valuable information for answering the widely debated questions to what extent the Eriksen flanker task and the Simon task differ, and how the differences determine the involved control mechanisms. Clearly, the two tasks differ in various aspects. The question is which one is crucial for their specific delta functions [9–11]. In the flanker task relevant and irrelevant information are of the same type and spatially separated, which allows, for instance, to control the effect of irrelevant information by spatial and categorical filtering [4]. In contrast, in the Simon task relevant and irrelevant information are of different type and are not spatially separated. Therefore, spatial filtering is hardly possible.

The important point, however, seems to be that these stimulus differences produce a different temporal distance between relevant and irrelevant response activation. Most researchers agree that stimulus location activates an associated response earlier than non-accidental stimulus features. As a result, the temporal distance between relevant and irrelevant activation is usually larger in the Simon task than in the flanker task. Our data support this idea and indicate that early irrelevant activation has to be suppressed or inhibited, which seems to be typical for the standard Simon task. However, it can also be induced for the flanker task by presenting the flankers ahead of the target. In this case, we also find negatively sloped delta functions. Our data further show that a certain temporal distance is necessary but not sufficient for obtaining negatively sloped delta functions. In our Simon-task experiment, where we modulated the slope by varying the proportion of congruent trials, the slope was positive or negative, depending on whether the proportion was high or low, respectively. Thus, our results demonstrate that the Simon task and the flanker task are rather similar in the sense that both can produced delta functions with different slopes.

Our results further show that suppression is not only an important control mechanism, but can also be applied rather flexibly. For a given task with specific stimulus and timing conditions, individuals can quickly adapt their mechanisms to the respective control demands. This is in line with other studies showing a high degree of flexibility in conflict control (e.g., [26, 62, 63]).

Finally, that suppression is crucial for obtaining negatively sloped delta functions is also confirmed by our modeling results. Despite their different architecture, both applied models had to assume suppression.

## References

[1] Stroop JR. Studies of interference in serial verbal reactions. Journal of Experimental Psychology. 1935;18:643–62.

[2] Steinhauser M, Hübner R. Distinguishing Response Conflict and Task Conflict in the Stroop Task: Evidence From Ex-Gaussian Distribution Analysis. Journal of Experimental Psychology: Human Perception and Performance. 2009;35(5):1398–412. 19803645 10.1037/a0016467

[3] Eriksen BA, Eriksen CW. Effects of noise letters upon the identification of a target letter in a nonsearch task. Perception & Psychophysics. 1974;16(1):143–9.

[4] Hübner R, Steinhauser M, Lehle C. A dual-stage two-phase model of selective attention. Psychological Review. 2010;117(3):759–84. 20658852 10.1037/a0019471

[5] Simon JR. Reactions toward the source of stimulation. Journal of Experimental Psychology. 1969;81(1):174–6. 5812172 10.1037/h0027448

[6] Proctor RW. Playing the Simon game: use of the Simon task for investigating human information processing. Acta Psychologica. 2011;136(2):182–8. 10.1016/j.actpsy.2010.06.010 20663486

[7] Hübner R, Mishra S. Evidence for strategic suppression of irrelevant activation in the Simon task. Acta Psychologica. 2013;144(1):166–72. 10.1016/j.actpsy.2013.05.012 23831497

[8] van den Wildenberg WPM, Wylie SA, Forstmann BU, Burle B, Hasbroucq T, Ridderinkhof KR. To head or to heed? Beyond the surface of selective action inhibition: a review. Frontiers in Human Neuroscience. 2010;4(December):222 10.3389/fnhum.2010.00222 21179583 PMC3004391

[9] Pratte MS, Rouder JN, Morey RD, Feng C. Exploring the differences in distributional properties between Stroop and Simon effects using delta plots. Attention, Perception, & Psychophysics. 2010;72(7):2013–25. 10.3758/APP.72.7.2013 20952797

[10] Ridderinkhof KR. Activation and suppression in conflict tasks: Empirical clarification through distributional analyses In: Prinz W, Hommel B, editors. Common Mechanisms in Perception and Action Attention & Performance, Vol XIX Oxford: Oxford University Press; 2002 p. 494–519.

[11] De Jong R, Liang CC, Lauber E. Conditional and unconditional automaticity: A dual-process model of effects of spatial stimulus-response correspondence. Journal of Experimental Psychology: Human Perception and Performance. 1994;20:731–50. 8083631 10.1037//0096-1523.20.4.731

[12] Gratton G, Coles MG, Donchin E. Optimizing the use of information: Strategic control of activation of responses. Journal of Experimental Psychology: General. 1992;121(4):480–506. . 1431740 10.1037//0096-3445.121.4.480

[13] Mansfield KL, van der Molen MW, Falkenstein M, van Boxtel GJ. Temporal dynamics of interference in Simon and Eriksen tasks considered within the context of a dual-process model. Brain and Cognition. 2013;82(3):353–63. 10.1016/j.bandc.2013.06.001 23856129

[14] Burle B, Spieser L, Servant M, Hasbroucq T. Distributional reaction time properties in the Eriksen task: marked differences or hidden similarities with the Simon task? Psychon Bull Rev. 2014;21:1003–10. 10.3758/s13423-013-0561-6 24302468 PMC4104006

[15] Ulrich R, Schröter H, Leuthold H, Birngruber T. Automatic and controlled stimulus processing in conflict tasks: Superimposed diffusion processes and delta functions. Cognitive psychology. 2015;78:148–74. 10.1016/j.cogpsych.2015.02.005 25909766

[16] Hommel B. The relationship between stimulus processing and response selection in the Simon task: Evidence for a temporal overlap. Psychological Research. 1993;55:280–90.

[17] Kornblum S, Hasbroucq T, Osman A. Dimensional overlap: Cognitive basis for stimulus-response compatibility—a model and taxonomy. Psychological Review. 1990;97(2):253–70. 2186425 10.1037/0033-295x.97.2.253

[18] Baroni G, Pellicano A, Lugli L, Nicoletti R, Proctor RW. Influence of temporal overlap on time course of the Simon effect. Experimental Psychology. 2012;59(2):88–98. 10.1027/1618-3169/a000130 22044788

[19] Burle B, Wildenberg van den WPM, Ridderinkhof KR. Dynamics of facilitation and interference in cue-priming and Simon tasks. European Journal of Cognitive Psychology. 2005;17(5):619–41.

[20] Hommel B. Spontaneous decay of response-code activation. Psychological Research. 1994;56(4):261–8. 8090861 10.1007/BF00419656

[21] Ridderinkhof KR. Micro- and macro-adjustments of task set: Activation and suppression in conflict tasks. Psychological Research. 2002;66:312–23. 10.1007/s00426-002-0104-7 12466928

[22] Eriksen CW. The flankers task and response competition: A useful tool for investigating a variaty of cognitive problems. Visual Cognition. 1995;2:101–18.

[23] Gratton G, Coles MGH, Sirevaag EJ, Eriksen CW, Donchin E. Pre- and poststimulus activation of response channels: A psychophysiological analysis. Journal of Experimental Psychology: Human Perception and Performance. 1988;14:331–44. 2971764 10.1037//0096-1523.14.3.331

[24] Flowers JH. Response priming effects in a digit naming task as a function of target-noise separation. Bulletin of the Psychonomic Society. 1980;16(6):443–6.

[25] Eriksen CW, Schultz DW. Information processing in visual search: A continuous flow conception and experimental results. Perception & Psychophysics. 1979;25(4):249–63. 461085 10.3758/bf03198804

[26] Wendt M, Kiesel A, Geringswald F, Purmann S, Fischer R. Attentional adjustment to conflict strength: evidence from the effects of manipulating flanker-target SOA on response times and prestimulus pupil size. Experimental Psychology. 2014;61(1):55 10.1027/1618-3169/a000227 24149239

[27] Mattler U. Delayed flanker effects on lateralized readiness potentials. Experimental Brain Research. 2003;151(2):272–88. 10.1007/s00221-003-1486-5 12739092

[28] Flowers JH, Wilcox N. The effect of flanking context on visual classification: The joint contribution of interactions at different processing levels. Perception & Psychophysics. 1982;32(6):581–91. 7167358 10.3758/bf03204214

[29] Greiner B. Subject pool recruitment procedures: Organizing experiments with ORSEE. Journal of the Economic Science Association. 2015;1(1):114–25. 10.1007/s40881-015-0004-4

[30] R Development Core Team R. A language and environment for statistical computing Vienna, Austria: R Foundation for Statistical Computing 2015;(Retrieved from http://www.r-project.org).

[31] Ratcliff R. Group reaction time distributions and an analysis of distribution statistics. Psychological Bulletin. 1979;86(3):446–61. 451109

[32] Hübner R, Töbel L. Does attentional selectivity in the flanker task improve discretely or gradually?. Frontiers in Psychology. 2012;3: 10.3389/fpsyg.2012.00434 23112779 PMC3481116

[33] White CN, Ratcliff R, Starns JJ. Diffusion models of the flanker task: Discrete versus gradual attentional selection. Cognitive Psychology. 2011;63:210–38. 10.1016/j.cogpsych.2011.08.001 . 21964663 PMC3195995

[34] Servant M, Montagnini A, Burle B. Conflict tasks and the diffusion framework: Insight in model constraints based on psychological laws. Cognitive Psychology. 2014;72:162–95. 10.1016/j.cogpsych.2014.03.002 24762975

[35] Hübner R. Does attentional selectivity in global/local processing improve discretely or gradually? Frontiers in Psychology. 2014;5:61 10.3389/fpsyg.2014.00061 24550875 PMC3909920

[36] White CN, Servant M, Logan GD. Testing the validity of conflict drift-diffusion models for use in estimating cognitive processes: A parameter-recovery study. Psychon Bull Rev. 2018;25:286–301. 10.3758/s13423-017-1271-2 28357629 PMC5788738

[37] Ratcliff R. A theory of memory retrieval. Psychological Review. 1978;85:59–108.

[38] Ratcliff R, Rouder JN. Modeling response times for two-choice decisions. Psychological Science. 1998;9(5):347–56.

[39] Brent RP. Algorithms for function minimization without derivatives. Englewood Cliffs, NJ: Prentice-Hall; 1973.

[40] Gegenfurtner KR. PRAXIS: Brent’s algorithm for function minimization. Behavior Research Methods, Instruments, and Computers. 1992;24:560–4.

[41] Powell MJ. An efficient method for finding the minimum of a function of several variables without calculating derivatives. The computer journal. 1964;7(2):155–62.

[42] Ratcliff R, Smith PL. A comparison of sequential sampling models for two-choice reaction time. Psychological Review. 2004;111:333–67. 15065913 10.1037/0033-295X.111.2.333 PMC1440925

[43] Schwarz G. Estimating the dimension of a model. The Annals of Statistics. 1978;6:461–4.

[44] Tipper SP. The negative priming effect: Inhibitory effects of ignored primes. Quarterly Journal of Experimental Psychology. 1985;37A:571–90. 10.1080/14640748508400920 4081101

[45] Frings C, Schneider KK, Fox E. The negative priming paradigm: An update and implications for selective attention. Psychon Bull Rev. 2015;22(6):1577–97. 10.3758/s13423-015-0841-4 25917144

[46] Neill WT, Kleinsmith AL. Spatial negative priming: Location or response? Attention, Perception, & Psychophysics. 2016;78(8):2411–9. 10.3758/s13414-016-1176-6 27465862

[47] D’Angelo MC, Thomson DR, Tipper SP, Milliken B. Negative priming 1985 to 2015: a measure of inhibition, the emergence of alternative accounts, and the multiple process challenge. The Quarterly Journal of Experimental Psychology. 2016;69(10):1890–909. 10.1080/17470218.2016.1173077 27065048

[48] Machado L, Guiney H, Struthers P. Identity-based inhibitory processing during focused attention. The Quarterly Journal of Experimental Psychology. 2013;66(1):138–59. 10.1080/17470218.2012.701651 22928521

[49] Hübner R, Lehle C. Strategies of flanker co-processing in single and dual tasks. Journal of Experimental Psychology: Human Perception and Performance. 2007;33:103–23. 10.1037/0096-1523.33.1.103 17311482

[50] Panis S, Schmidt T. What is shaping RT and accuracy distributions? Active and selective response inhibition causes the negative compatibility effect. Journal of Cognitive Neuroscience. 2016. 10.1162/jocn_a_00998 27315271

[51] Sumner P. Negative and positive masked-priming–implications for motor inhibition. Advances in Cognitive Psychology. 2007;3(1–2):317–26. 10.2478/v10053-008-0033-0 PMC2864966 20517517

[52] Hasegawa K, Takahashi Sy. Functional difference between sustained and transient modulations of cognitive control in the Simon Task: Evidence from false alarm responses on no-go trials. PLoS One. 2013;8(11):e81804 10.1371/journal.pone.0081804 24303072 PMC3841129

[53] Stürmer B, Leuthold H, Soetens E, Schröter H, Sommer W. Control over location-based response activation in the Simon task: behavioral and electrophysiological evidence. Journal of Experimental Psychology: Human Perception and Performance. 2002;28(6):1345–63. 12542132 10.1037//0096-1523.28.6.1345

[54] Toth JP, Levine B, Stuss DT, Oh A, Winocur G, Meiran N. Dissociation of processes underlying spatial SR compatibility: Evidence for the independent influence of what and where. Consciousness and cognition. 1995;4(4):483–501. 10.1006/ccog.1995.1052 8750420

[55] Torres-Quesada M, Funes MJ, Lupiáñez J. Dissociating proportion congruent and conflict adaptation effects in a Simon–Stroop procedure. Acta psychologica. 2014;142(2):203–10. 10.1016/j.actpsy.2012.11.015 23337083

[56] Töbel L, Hübner R, Stürmer B. Suppression of irrelevant activation in the horizontal and vertical Simon task differs quantitatively not qualitatively. Acta Psychologica. 2014;152(0):47–55. 10.1016/j.actpsy.2014.07.007 25113126

[57] Akçay Ç, Hazeltine E. Conflict monitoring and feature overlap: Two sources of sequential modulations. Psychon Bull Rev. 2007;14(4):742–8. 17972743 10.3758/bf03196831

[58] Taylor DA. Time course of context effects. Journal of Experimental Psychology: General. 1977;106(4):404.

[59] Proctor RW, Miles JD, Baroni G. Reaction time distribution analysis of spatial correspondence effects. Psychon Bull Rev. 2011;18:242–66. 10.3758/s13423-011-0053-5 21327376

[60] Vorberg D, Mattler U, Heinecke A, Schmidt T, Schwarzbach J. Different time course for visual perception and action priming. Proc Natl Acad Sci U S A. 2003;100:6275–80. 10.1073/pnas.0931489100 12719543 PMC156362

[61] Schwarz W, Miller J. Response time models of delta plots with negative-going slopes. Psychon Bull Rev. 2012;19(4):555–74. 10.3758/s13423-012-0254-6 22610358

[62] Jost K, Wendt M, Luna-Rodriguez A, Löw A, Jacobsen T. Strategic control over extent and timing of distractor-based response activation. Journal of Experimental Psychology: Learning, Memory, and Cognition. 2017;43(2):326 27732019 10.1037/xlm0000326

[63] Gehring WJ, Gratton G, Coles MGH, Donchin E. Probability effects on stimulus evaluation and response processes. Journal of Experimental Psychology: Human Perception and Performance. 1992;18:198–216. 1532188 10.1037/0096-1523.18.1.198

